# Decomposition profile data analysis of multiple drug effects identifies endoplasmic reticulum stress-inducing ability as an unrecognized factor

**DOI:** 10.1038/s41598-020-70140-9

**Published:** 2020-08-04

**Authors:** Katsuhisa Morita, Tadahaya Mizuno, Hiroyuki Kusuhara

**Affiliations:** Graduate School of Pharmaceutical Sciences, The University of Tokyo, Bunkyo-ku, Tokyo, 113-0033 Japan

**Keywords:** Chemical biology, Computational biology and bioinformatics

## Abstract

Chemicals have multiple effects in biological systems. Because their on-target effects dominate the output, their off-target effects are often overlooked and can sometimes cause dangerous adverse events. Recently, we developed a novel decomposition profile data analysis method, orthogonal linear separation analysis (OLSA), to analyse multiple effects. In this study, we tested whether OLSA identified the ability of drugs to induce endoplasmic reticulum (ER) stress as a previously unrecognized factor. After analysing the transcriptome profiles of MCF7 cells treated with different chemicals, we focused on a vector characterized by well-known ER stress inducers, such as ciclosporin A. We selected five drugs predicted to be unrecognized ER stress inducers, based on their inducing ability scores derived from OLSA. These drugs actually induced X-box binding protein 1 splicing, an indicator of ER stress, in MCF7 cells in a concentration-dependent manner. Two structurally different representatives of the five test compounds exhibited similar results in HepG2 and HuH7 cells, but not in PXB primary hepatocytes derived from human-liver chimeric mice. These results indicate that our decomposition strategy using OLSA uncovered the ER stress-inducing ability of drugs as an unrecognized effect, the manifestation of which depended on the background of the cells.

## Introduction

Small compounds have the capacity to interact with multiple cellular proteins, thereby mediating multiple effects depending on their exposure to interacting proteins. It is quite difficult to identify the full range of effects of a new chemical, even for its developers, and some factors may be unrecognized. Even approved drugs show off-target effects that are often unrecognized during drug development, and which can lead to adverse reactions including lethal effects. For instance, astemizole has been withdrawn from the market in most countries because it causes a fatal side effect, cardiac arrhythmia^1,2^, by blocking the human Ether-a-go-go Related Gene potassium channel, thereby causing QT interval prolongation and torsade de pointes. However, we should not be too negative about unrecognized effects of small compounds, because identification of these can lead to drug repurposing. For example, memantine was synthesized in 1968 as a derivative of amantadine, an anti-influenza agent. Recently, it was revealed that this anti-influenza agent is also an antagonist for the N-methyl-D-aspartate receptor, and the drug has become a medication for dementia^3^. Thus, the important question is how such unrecognized effects of chemicals can be identified.

Profile data analysis is quite useful in addressing this issue. Although the terminology sometimes varies between contexts, the method is a type of multivariate analysis that describes data using multiple variables and investigates the relationship between data with high robustness and detection power by sharing information about the structure of the data^4^. Data from “omics” analysis, which converts biological information of a specimen into numerical information with its comprehensiveness, is an example of profile data analysis.

Connectivity Map (CMap), created by the Broad Institute, was a pioneer of profile data analysis. The project collected more than 6,000 microarray data investigating the cellular responses of cultured cells treated with 1,309 low-molecular-weight compounds and defined “gene signatures” as those genes up- and down-regulated by each compound. These “big data” have been utilized to search for compounds that induce similar or even counteracting effects on gene expression^5^. Such gene expression changes comprise a profile and have, for example, been employed to identify that ribavirin could induce reprogramming of docetaxel-resistant prostate cancer to be docetaxel sensitive^6^.

Recently, we have developed a novel profile data analysis method, orthogonal linear separation analysis (OLSA)^7^. The method was designed to allow separation of multiple effects of small compounds by factor analysis-based matrix decomposition, assuming that the decomposition process helps to understand the mode of action (MOA) of the chemicals. In fact, the outputs, latent variables expressed as vectors, correspond to consistent biological outcomes. This has contributed to identifying novel effects of some chemicals, such as the induction of autophagy^7^.

Using the concepts of OLSA, i.e., decomposition and understanding, we hypothesized that it was a suitable strategy to identify the unrecognized effects of chemicals. In this study, we focused on the ability of drugs to induce endoplasmic reticulum (ER) stress. The ER is an organelle that governs protein synthesis and folding. When a proportion of newly synthesized proteins are not appropriately folded and accumulate in the ER, cells employ a signalling pathway called the unfolded protein response (UPR) to manage the stress^8^. However, some drugs overwhelm this compensatory machinery and cause adverse events such as drug-induced liver injury (DILI)^9^. Here, we provide the evidence that the analysis of multiple effects of drugs using profile data analysis can detect the ability of drugs to induce ER stress as an unrecognized effect that cannot be addressed by conventional multivariate analyses.

## Results

### Characterization of a vector focussed on the ability of chemicals to induce ER stress

**Figure 1:**
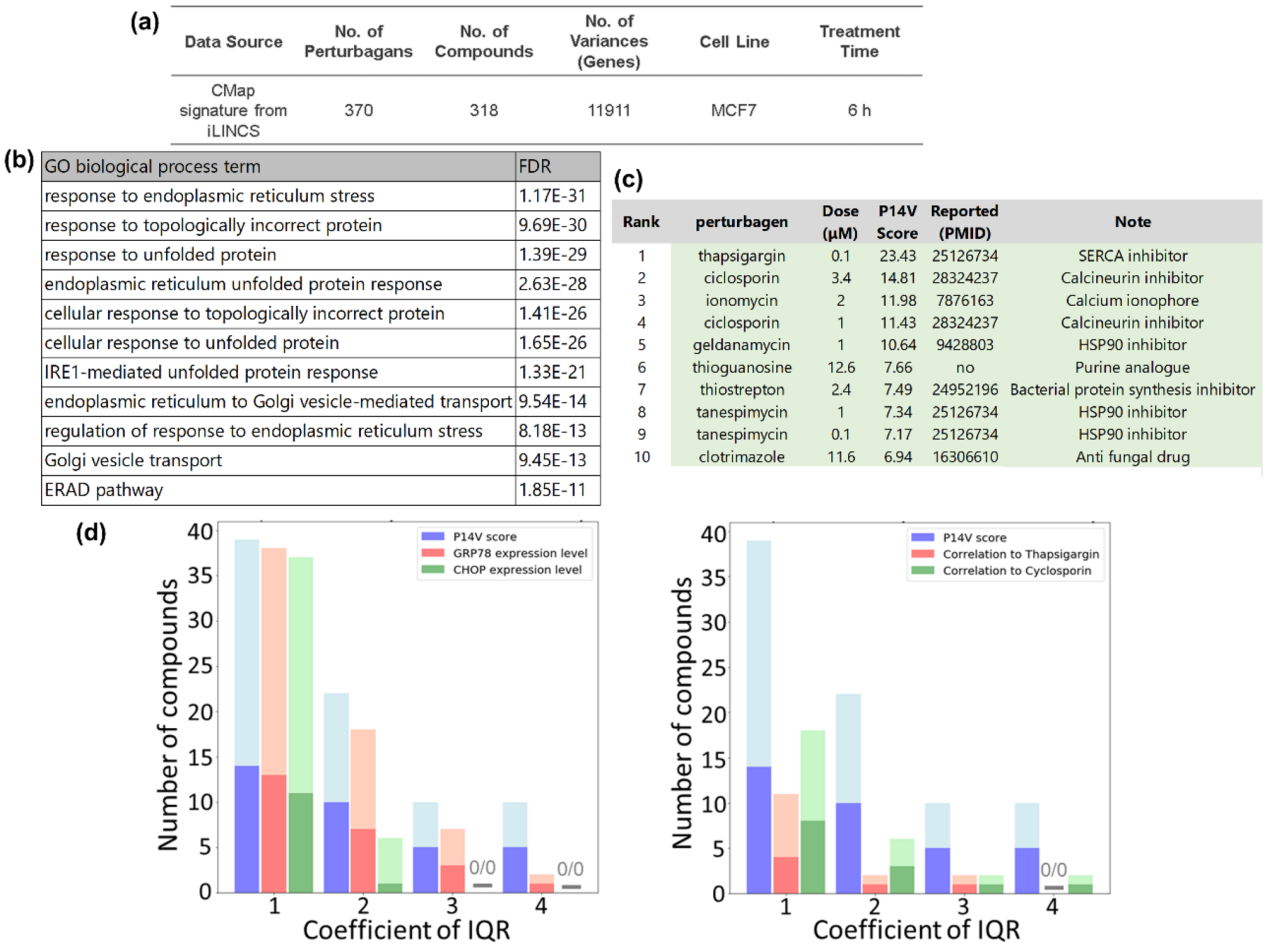
Characterization of a vector contracting the ER stress inducing ability of chemicals. (**a**) Summary of data analysed in this study. Information about the transcriptome data subjected to OLSA. (**b**) Result of the GO analysis of P14V. The top 119 genes (1% of total) constituting P14V were subjected to GO analysis (biological process) using the Enrichment analysis of the Gene Ontology Consortium (https://geneontology.org/). P values were calculated with Fisher’s exact test and adjusted for false discovery rate (FDR, Benjamini–Hochberg method). GO terms are given in descending order according to each FDR. GO terms with FDR < 10^–11^ are shown. (**c**) List of the compounds with a high P14V score. Compounds are sorted by each P14V score and the top 10 compounds are listed in a table. PubMed was searched for publications related to ER stress or UPR using (“ER stress” OR “UPR”) AND each compound name. (**d**) Comparison of the number of hit chemicals detected. The numbers of extracted compounds that fit the criteria of median plus the indicated IQR of P14V score, GRP78 or CHOP mRNA expression, and Pearson correlation with thapsigargin or ciclosporin A were compared and visualized as bars corresponding to the numbers of publications related to ER stress or UPR. The dark and light colours indicate the compounds with and without publications, respectively.

From the CMap database, we obtained transcriptome data describing the cellular responses of MCF7 cells treated with 318 compounds and subjected these data to OLSA (Fig. 1a). To select a vector that was related to toxic aspects of drugs, we analysed the component genes and the characteristic compounds for each vector. The genes constituting each vector were sorted by their absolute values and the top 1% genes were subjected to gene ontology (GO) analysis. Hereafter, we use the term P*X*V to indicate the Principal *X*^th^ Vector component (*X* means the rank of the contribution). Based on these results, we focused on vector P14V because the top 1% of vector genes was significantly enriched for GO terms relevant to ER stress induction, such as 0034976, 0035966, and 0006986 (Fig. 1b, Supplementary Data). The ER stress response is well-known to be involved in DILI, which is often the cause for drugs being withdrawn from the market and is a precise toxic effect of some drugs^10–12^. In OLSA, the scores calculated for a vector indicate the strength of the effect represented by the vector. Many of the drugs and compounds with high P14V scores (i.e., a strong P14V-related effect) were previously reported to be associated with the UPR or ER stress (Fig. 1c). Consistency between the component genes and the characteristic compounds of P14V with respect to ER stress motivated us to investigate this vector as a possible detector of latent aspects of drugs.

It was important to emphasize the differences between the scores defined by the focused vector and those identified by other generally used indicators, such as the expression levels of well-known ER stress markers and similarity to well-known ER stress inducers. Therefore, we surveyed the literature to clarify the ability of high-scoring compounds to induce ER stress, as defined by the indicated interquartile range (IQR) of the score distribution of each indicator (Supplementary Fig. S1). As shown in Fig. 1d, the number of previously confirmed ER stress inducers within the compounds with a high P14V score was relatively high compared with those determined by other well-known markers or inducers, particularly at high IQR, although the reported ratio was comparable for all indicators. Note that the lack of a relevant publication does not mean that a compound does not induce ER stress, just that no information is available. All of these results indicate that the features of P14V response scores are different from those calculated by conventional approaches and are suitable for exploratory use in the detection of the ability of chemicals to induce ER stress.

### Selection of test drugs and decomposition analysis

**Figure 2:**
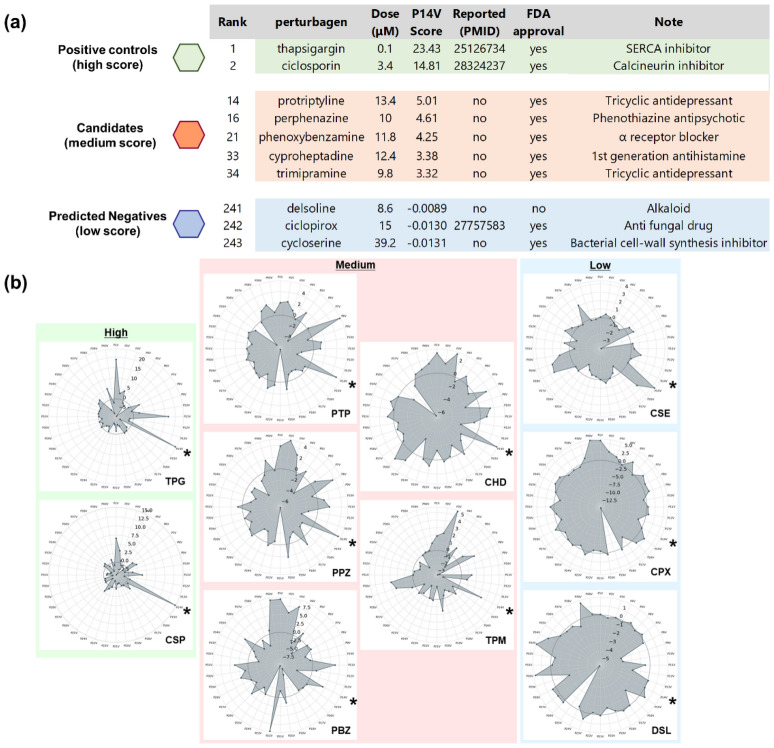
Selection of test drugs and decomposition analysis. (**a**) List of the candidate drugs and control chemicals. Candidate drugs were selected and listed based on the following criteria: (1) drugs with a moderate score defined as median plus 0.75 times IQR of P14V score distribution, (2) no publications related to ER stress or UPR stress, and (3) drugs approved by FDA for oral use. Three compounds with low absolute values for P14V score (the bottom three compounds) were also selected as predicted negative compounds. (**b**) Decomposition analysis of each chemical. Radar chart visualizing OLSA outputs of the selected compounds. Direction indicates each vector score. Central black line is the line representing zero score. TPG, thapsigargin; CSP, ciclosporin A; PTP, protriptyline; PPZ, perphenazine; PBZ, phenoxybenzamine; CHD, cyproheptadine; TPM, trimipramine; DSL, delsoline; CPX, ciclopirox; CSE, cycloserine. Vectors are arranged clockwise with the top being P1V. *, P14V.

Unlike the compounds with high P14V scores, some of the compounds with moderate scores had not previously been associated with ER stress (Fig. 2a). We chose five such drugs (protriptyline, perphenazine, phenoxybenzamine, cyproheptadine, and trimipramine) as the candidate drugs for validation based on the following criteria: (1) drugs with a moderate score defined as median plus 0.75 times the IQR of the P14V score distribution, (2) no publications related to ER stress or UPR stress, and (3) drugs approved by the US Food and Drug Administration (FDA) for oral use (Supplementary Fig. S2). Three compounds with a low absolute value for P14V score (bottom three compounds, cycloserine, ciclopirox, and delsoline) were also selected as predicted negative compounds. All the candidate drugs clustered in different clusters from that, including the well-known ER stress inducers thapsigargin and ciclosporin A (Supplementary Fig. S3). Of note, phenoxybenzamine and trimipramine also clustered distantly from the well-known inducers, which indicates that these drugs cannot be listed using representative clustering methods.

Visualization of the response scores of a small compound provided us with valuable information for clarification of the full range of effects of the chemical. To obtain insight for each candidate, we plotted the response scores of test compounds. The P14V scores of thapsigargin and ciclosporin A were the highest and were strikingly high compared with those of the other vectors, consistent with the fact that these are well-known ER stress inducers (Fig. 2b). However, the P14V scores of the candidate drugs were comparable to those for other high-scoring vectors, although, except for cyproheptadine, these P14V scores were not the highest of all vectors. These results suggest that the capacity of the five candidates to induce ER stress is latent, and that decomposition analysis makes it easy to recognize this inductive ability.

### Induction of ER stress in MCF7 cells by test compounds

**Figure 3:**
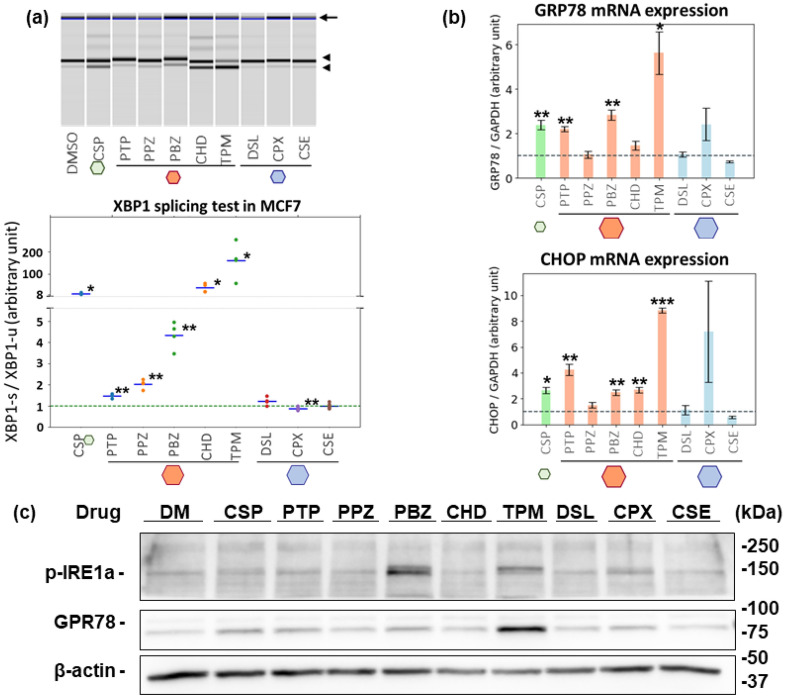
Induction of ER stress in MCF7 cells by test compounds. (**a**) XBP1 splicing test of MCF7 cells treated with the candidate drugs. MCF7 cells were treated with the candidate drugs for 6 h at X-times the concentration used for the acquisition of transcriptome data. X: CSP, 1; PTP, 3; PPZ, 3; PBZ, 10; CHD, 10; TPM, 10; DSL, 10; CSP, 10; CSE, 10. cDNA was synthesized from mRNA and subjected to conventional PCR. The concentrations of spliced and unspliced XBP1 amplified products were quantified using a MultiNA electrophoresis apparatus. The arrow, the upper arrowhead, and the lower arrowhead indicate a non-specific band, the unspliced XBP1, and the spliced XBP1, respectively. Each value in the lower graph indicates a ratio of spliced to unspliced XBP1 concentration. Note that TPG was excluded from these experiments because it exhibited quite a high value and was not suitable as a positive control in data visualization. (**b**) Changes in expression of mRNA for ER stress markers induced by the candidate drugs. MCF7 cells were treated with the candidate drugs for 24 h at X-times the concentration used for transcriptome data acquisition. X: CSP, 1; PTP, 2; PPZ, 1; PBZ, 10; CHD, 3; TPM, 5; DSL, 10; CPX, 10; CSE, 10. cDNA was synthesized from mRNA and subjected to quantitative PCR analysis. (**c**) Changes in protein expression of ER stress markers induced by the candidate drugs. MCF7 cells were treated with the candidate drugs for 24 h at X-times the concentration used for transcriptome data acquisition. X: CSP, 1; PTP, 2; PPZ, 1; PBZ, 10; CHD, 3; TPM, 5; DSL, 10; CPX, 10; CSE, 10. The whole-cell lysate was analysed by western blotting. A representative result of at least two independent experiments is shown in each figure. All significance tests were conducted with Welch *t* test, *P* values were adjusted for FDR (Benjamini–Hochberg method), and only significant differences between DMSO and the tested compounds are shown: *P < 0.05, **P < 0.01, ***P < 0.001.

We hypothesized that the candidate drugs (five drugs with moderate P14V scores) had a latent ability to induce ER stress at high concentrations while the three predicted negative compounds (P14V scores close to zero) did not. To verify this hypothesis, we investigated the non-canonical splicing of X-box binding protein 1 (XBP1). Under ER stress, XBP1 is non-canonically spliced to its active form by inositol-requiring enzyme 1 α (IRE1α) in the cytosol, translocates into the nucleus, and functions as a transcription factor; therefore, the ratio of spliced to unspliced XBP1 is a good indicator of ER stress^13,14^. We have established a system to evaluate XBP1 splicing using MultiNA, an electrophoresis apparatus that incorporates a microchip. This assay system demonstrated a dose-dependent increase in the XBP1 splicing ratio caused by a well-known ER stress inducer, ciclosporin A (Supplementary Fig. S4). MCF7 cells were treated with the test compounds at various concentrations for 6 h and XBP1 splicing ratios were measured. Although the cells were adherent and active at all concentrations, the morphology of the cells became rounded (early phenotype of apoptosis) under some conditions (Supplementary Fig. S5). As shown in Fig. 3a and Supplementary Fig. S6, all the candidate compounds enhanced XBP1 splicing at the highest concentration while the predicted negative compounds did not. Although the magnitude of the increase varied between the drugs, a trend toward a concentration-dependent increase was observed. We also investigated the effects of these drugs on the expression levels of ER stress markers in MCF7 cells. Transcripts of *GRP78* and *CHOP*, well-known ER stress markers^15,16^, were quantified using quantitative polymerase chain reaction (qPCR,Fig. 3b). Three of the five candidates (protryptiline, phenoxybenzamine, and trimipramine) significantly increased the levels of mRNA for both markers, while cyproheptadine enhanced only *CHOP* and perphenazine had no effect on either marker. Western blotting analysis showed that phenoxybenzamine and trimipramine induced a clear increase in expression of GRP78 and phosphorylated-IRE1α while the other candidate compounds had only small effects (Fig. 3c). These results indicate that the vector signature generated using OLSA detected the latent ability of the candidate drugs to induce ER stress, although the extent of the effects did not correlate with their scores for the signature and varied among the candidate drugs.

### Induction of ER stress in hepatic cancer-derived cell lines by representative test compounds

The results using MCF7 cells supported that OLSA-based detection of latent ER stress-inducing ability worked well in the cell line from which the transcriptome profiles were obtained. We were interested to determine whether this was also true in other cell lines. Because ER stress is one of the major causes of DILI, we selected the hepatic cancer-derived cell lines, HepG2 and HuH7. Because of the scale of the experiments, only two representative candidate drugs, trimipramine and phenoxybenzamine, were subjected to these further analyses. These two drugs were chosen based on their distances from well-known ER inducers and their structural dissimilarity. As shown in Supplementary Fig. S3, these two drugs both clustered distantly from both thapsigargin and ciclosporin A. In addition, while protriptyline, perphenazine, cyproheptadine, and trimipramine share a similar 3-ring structure, phenoxybenzamine does not (Supplementary Fig. S2).

**Figure 4:**
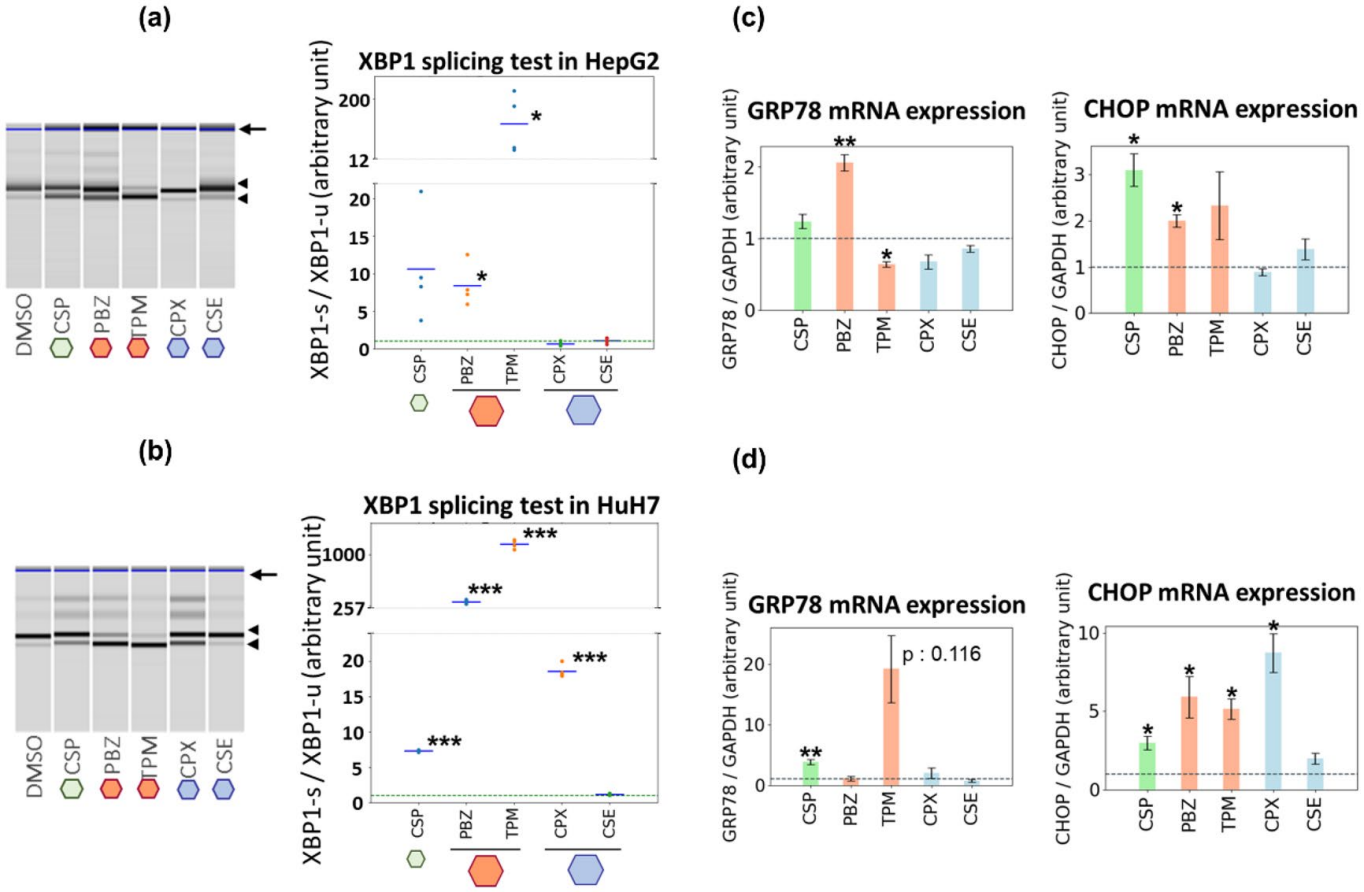
Induction of ER stress in hepatic cancer-derived cell lines by representative test compounds. (**a**,**b**) XBP1 splicing test of HepG2 and HuH7 cells treated with the representative drugs. HepG2 and HuH7 cells were treated with the candidate drugs for 6 h at X-times the concentration used for transcriptome data acquisition. X: CSP, 1; PBZ, 10; TPM, 10; CPX, 10; CSE, 10. cDNA was synthesized from mRNA and subjected to conventional PCR. The concentrations of spliced and unspliced XBP1 amplified products were quantified using a MultiNA electrophoresis apparatus. The arrow, the upper arrowhead, and the lower arrowhead indicate a non-specific band, the unspliced XBP1, and the spliced XBP1, respectively. Each value in the lower graph indicates a ratio of spliced to unspliced XBP1. (**c**,**d**). Changes in expression of mRNA for ER stress markers in HepG2 and HuH7 cells. HepG2 and HuH7 cells were treated with the candidate drugs for 24 h at X-times the concentration used for transcriptome data acquisition. X: CSP, 1; PBZ, 3; TPM, 3; CPX, 10; CSE, 10. cDNA was synthesized from mRNA and subjected to quantitative PCR analysis. A representative result of at least two independent experiments is shown in each figure. All significance tests were conducted using Welch’s *t* test, *P* values were adjusted for FDR (Benjamini–Hochberg method), and only significant differences between DMSO and the tested compounds are shown: *P < 0.05, **P < 0.01, ***P < 0.001.

First, we checked the effects of the representative drugs on the morphology of the two liver-derived cell lines. Interestingly, the morphology of both cell lines was much more strongly affected than that of MCF7 cells, and both HepG2 cells and HuH7 cells were nearly all dead at the highest drug concentration used in MCF7 cells. This suggests that there are differences between cell lines in their morphological responses to, and tolerated dose of, trimipramine and phenoxybenzamine (Supplementary Fig. S7a). Next, we treated both HepG2 cells and HuH7 cells with the representative drugs for 6 h at a concentration below the highest concentration employed in the experiments with MCF7 cells, and we evaluated the non-canonical splicing of XBP1 and the morphology of the two cell lines (Supplementary Fig. S7b). Figure 4a,b demonstrates that XBP1 splicing was significantly increased in both cell lines by the representative drug treatments. Cycloserine, the compound with the second-lowest score, had no effect on the splicing ratio in either cell line, while ciclopirox, which had the third-lowest score, significantly increased the splicing in HuH7 cells at high concentrations, but did not affect it in HepG2 cells (Supplementary Figs S8 and S9). Consistent with these findings, we confirmed a similar trend in the changes in expression of *GRP78* and *CHOP* (Fig. 4c,d). These combined results indicate that, with the exception of ciclopirox in HuH7 cells, the latent ER stress-inducing ability predicted by OLSA is close to that observed in cell lines other than the line utilized in the acquisition of the transcriptome profiles, although the extent of the latent effect varies among cell lines.

### Induction of ER stress in PXB cells by representative test compounds

It is widely accepted that the function of metabolic enzymes such as cytochrome P450 (CYP) is impaired in tumour-derived cell lines^17^. Thus, the manifestations of ER stress induced by the candidate drugs may be different in normal cells, which motivated us to test whether the latent effect could be observed in primary cultured cells. Because the liver is the main organ that deals with xenobiotics such as drugs and chemicals, several liver-derived resources have been developed for evaluation of the activities of drug transporters and metabolic enzymes. PXB cells, primary cultured hepatocytes derived from human-liver chimeric mice, are one such state-of-the-art resource^18^. Their normal metabolic activities and reactivity to compounds are relatively well preserved compared with those of hepatic cancer-derived cell lines such as HepG2 and HuH7, although it is well known that metabolic activity rapidly decreases in primary cultured cells after seeding^19^.

**Figure 5:**
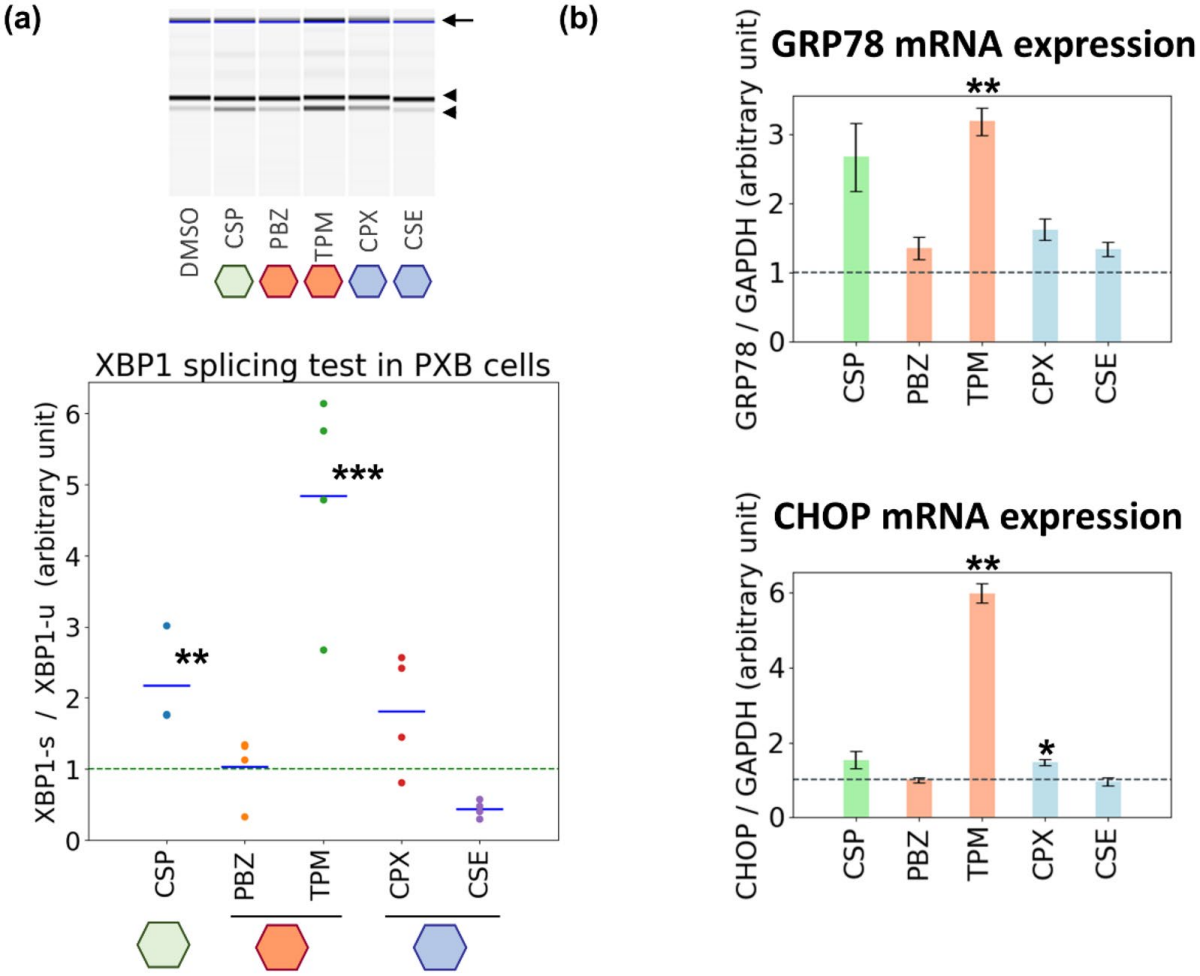
Induction of ER stress in PXB cells by representative test compounds. (**a**) XBP1 splicing test of PXB cells treated with the representative drugs. PXB cells were treated with the representative drugs for 24 h at X-times the concentration used for transcriptome data acquisition. X: CSP, 1; PBZ, 10; TPM, 10; CPX, 10; CSE, 10. cDNA was synthesized from mRNA and subjected to conventional PCR. The concentrations of spliced and unspliced XBP1 amplified products were quantified using a MultiNA electrophoresis apparatus. The arrow, the upper arrowhead, and the lower arrowhead indicate a non-specific band, the unspliced XBP1, and the spliced XBP1, respectively. Each value in the lower graph indicates a ratio of spliced to unspliced XBP1. (**b**). Changes in expression of mRNA for ER stress markers. Extracted cDNA as described in (**a**) was subjected to quantitative PCR analysis. A representative result of at least two independent experiments is shown in each figure. All significance tests were conducted using Welch’s *t* test, *P* values were adjusted for FDR (Benjamini–Hochberg method), and only significant differences between DMSO and the tested compounds are shown: *P < 0.05, **P < 0.01, ***P < 0.001.

We employed PXB cells to address the above question by evaluating the effects in these cells of the representative candidate compounds, trimipramine and phenoxybenzamine. Of note, the morphology of the cells was almost unaffected by treatment with these compounds at the highest concentration used in MCF7 cells, which severely damaged the two hepatic cancer-derived cell lines, HepG2 and HuH7 (Supplementary Fig. S10). PXB cells were treated with trimipramine and phenoxybenzamine and two of the predicted negative compounds, cycloserine and ciclopirox. After 24-h treatment, non-canonical splicing of XBP1 was clearly increased in the cells treated with trimipramine although the increase was relatively small compared with that observed in the other cell lines (Fig. 5a). Of note, neither phenoxybenzamine nor the predicted negative compounds affected the splicing ratio. With respect to mRNA expression, trimipramine significantly enhanced levels of mRNA for the ER stress markers *GRP78* and *CHOP* (Fig. 5b). The other compounds had almost no effect on these markers, although ciclopirox induced a small but significant enhancement of *CHOP* expression, similar to the trend observed in HuH7 cells (Fig. 4d). These results indicate that in some instances, the prediction of the latent ER stress-inducing ability of drugs by analysing transcriptome data derived from tumour cell lines can be extrapolated to primary cultured hepatocytes (true for one of two drugs under the experimental conditions used in this study).

## Discussion

Because a small compound interacts not only with its primary target but also with multiple secondary targets, we often observe unexpected effects of chemicals on cellular functions. These unrecognized effects of small compounds are sometimes beneficial, but can also be harmful. The best solution to this problem would be to define the full range of effects of a chemical. However, this is quite difficult because, in most cases, the “full range” remains undefined. To address this issue, we recently developed a new method of profile data analysis, OLSA, to facilitate understanding of the characteristics of a chemical by decomposing its effects represented by a genetic profile^7^. One example of a damaging unrecognized effect is an adverse event related to drug use. In the present study, we investigated whether the ability of a compound to induce ER stress, one of the major causes of adverse events, can be detected by our “decomposition and understanding” strategy, even when this ability is weak and latent.

In this study, we confirmed that all the candidate drugs had the latent ability to induce ER stress as defined by non-canonical splicing of XBP1^13,14^, in MCF7 cells, in which the analysed transcriptome data were obtained (Fig. 3). However, some of the drugs did not enhance the expression of marker genes or proteins. One of the possible explanations for this is the differences in the time course of these experiments. The expression levels of the markers were measured after 24-h treatment with the candidate compounds to maximize detection sensitivity, while evaluation of XBP1 splicing was performed after a 6-h treatment^20–22^. This short treatment duration is suitable for the evaluation and description of the effects of a drug because longer-term treatment can result in feedback reactions and blunt the effects of interest. In fact, the transcriptome profile data in CMap was mainly acquired after 6 h of compound treatment^5^. An important future task would be to investigate the features of these drugs at other time points. Note that this short treatment duration in MCF7 cells, and probably in other cell lines as well, is enough to induce the expression changes of ER stress-related genes, although the changes are too small to be recognized from a macro perspective in the transcriptome.

As shown in Supplementary Fig. S3, phenoxybenzamine and trimipramine located into completely different clusters from those containing the two well-known ER stress inducers. This indicated that this unrecognized effect of these drugs could not be determined using the Ward method, a representative method of unsupervised multivariate analysis^23,24^. Because many of the clustering methods utilize whole variables, in practice the results tend to be dominated by the on-target effects and not to reflect the off-target effects. In contrast, as shown in Fig. 2b, our “decomposition and understanding” strategy using OLSA is effective for the evaluation of such off-target effects.

As shown in Fig. 3, we could confirm the induction of ER stress by the test drugs at concentrations higher than those employed in the original acquisition of transcriptome data. Note also that the actual exposure concentration may vary between this study and the original transcriptome data acquisition, because there may have been undefined differences in the experimental conditions, such as the batch of foetal bovine serum (FBS). However, compared with the test drugs, the predicted negative compounds did not induce ER stress, even at the highest concentration used. Total strength (L2 norm of gene expression changes of each sample) of negative compounds is similar to that of positive compounds (Supplementary Data). Considering that total strength represents the magnitude of drug effects, treatment with the negative compounds at the CMap concentrations, which are the base concentrations, have enough effectivity to be compared with each other^7,25,26^. These results indicated that the compounds with moderate scores on the ER stress vector potentially have ER stress-inducing ability, which, depending on the exposure concentration, can be latent. It should be kept in mind that in clinical practice, the exposure concentration can be unexpectedly high because of many unintended differences between patients such as mutations, drug–drug interactions, and tissue injury^27–29^.

The successful validation of our method in MCF7 cells means that our approach worked well in identifying whether a chemical has the potency to cause the effect of interest. In this study, we also investigated the effect in other cell lines. As shown in Fig. 5^,^ PXB cells exhibited no ER stress responses to phenoxybenzamine, which induced ER stress in all the other cell lines. Note that knowing the potency of a chemical is a totally different issue to prediction of the nature and extent of the effect, particularly in different environments. The factors that affect the manifestation of an effect by a chemical are mainly divided into two types: dynamic factors, such as the affinity of a chemical for the target protein, which determine the magnitude of the effect, and kinetic factors, which regulate the exposure of the chemical to the target. To discuss the former, we should know the target of each chemical, although it is quite difficult to speculate about the direct target molecule only from transcriptome data. For the latter, one of the possible explanations for the specificity of the PXB cell phenotype is a difference in metabolic enzymes. PXB cells are human primary hepatocytes derived from human-liver chimeric mice, and it is well-known that metabolic enzymes such as CYP are downregulated in tumour-derived cell lines compared with primary culture cells^17^. Indeed, differential CYP expression profiles were confirmed by the analyses of transcriptome data comparing MCF7 cells, HepG2 cells, HuH7 cells, and human hepatocytes (Supplementary Fig. S11). Note that we analysed human hepatocytes instead of PXB cells because we could not obtain transcriptome data for PXB cells. These findings are consistent with the relatively low level of XBP1 splicing induced by trimipramine in PXB cells, because the drug is mainly metabolized by CYP2D6, which is expressed at a higher level in human hepatocytes than in the other cell types^30^ (Supplementary Fig. S12). Because of a lack of information about the metabolic pathways for phenoxybenzamine, the ADMET Predictor was employed to investigate the metabolic enzymes, with the results showing that the relevant enzymes such as CYP1A2 are similarly highly expressed in PXB cells^31^. Of course, from these expression data we can analyse only correlations,further studies are necessary to identify the cause of the different phenotype of phenoxybenzamine treatment from the viewpoint of exposure. With regard to the dynamic factors, limited information is so far available for phenoxybenzamine^32,33^. It will be helpful to employ chemoinformatics methods such as docking and simulation in studies to survey dynamic factors, which is an important future task in this field^34^.

Is it possible to speculate about the nature of the pathways regulating latent ER stress inducing ability? First, western blotting analysis revealed an increase in phosphorylated IRE1, which is upstream of XBP1. This result indicated that these drugs activate IRE1–XBP1 signalling, the most preserved of the three well-known ER stress signalling pathways (IRE1–XBP1 signalling, activating transcription factor 6 signalling, and (PKR)-like ER kinase signalling)^35,36^ (Fig. 3c). Next, what about upstream of IRE1–XBP1? We would like to suggest that the response scores of the other vectors help us to answer this question. Figure 2b and the Supplementary Data show that although the P14V scores of the candidates were similar, their scores for other vectors varied. Phenoxybenzamine and trimipramine, which clustered differently and have different structures, exhibited relatively large differences in scores for some vectors such as P18V, P19V, P22V, and P23V. Interestingly, combinatorial treatment with these two drugs resulted in synergistic enhancement of XBP1 splicing, which supports the hypothesis that they act at different points (Supplementary Fig. S13). It is also noteworthy that ciclopirox, which induced biphasic and strange XBP1 splicing profiles in MCF7 cells and HuH7 cells although its P14V score was zero, had a strikingly high P19V score. Because OLSA contracts dimensions based on a linear hypothesis, one of the strong points of the method is the ease with which biological understanding of the meaning of the new indicators could be described as a weighted gene group, although non-linear approaches provide superior resolution^7^. For instance, the main component genes of the vectors distinguishing phenoxybenzamine and trimipramine are significantly associated with particular GO terms, which may be clues to investigate their molecular entities (Supplementary Data). We have no clear explanation for the differences between test drugs or the peculiarity of ciclopirox, but consider that the elucidation of these mechanisms was not the main objective of this study. However, to achieve not just an understanding of the effects of a chemical but to be able to predict the way in which they are manifested, it would be important to test whether the molecular-biology-friendly features of OLSA contribute to identifying the upstream pathways.

We would like to suggest that unrecognized effects of chemicals may be a novel source of drug discovery. In CMap, one of the most successful in silico platforms for drug repositioning^6,37^, the “entire” features of each compound could be classified by the differentially expressed genes that were significantly affected by the dominant, mainly on-target, effects. In contrast, our decomposition strategy provides an alternative approach that turns the focus to the off-target effects. Although such effects are often weak and can be difficult to be directly repurposed as therapeutics, they do exist as biological responses and have the potential to be drug targets for diseases associated with those responses.

Shedding light on the unrecognized effects of chemicals using OLSA may also be useful in exploring biology, because such chemicals have contributed to the development of biology; chemical biology. For instance, necrostatin-1, an inhibitor of necroptosis, was utilized to characterize an important cell-death mechanism by targeting an at first unknown protein; two years later the target was identified as a key component of necroptosis^38,39^. Thus, the uncharacterized effects of small compounds are useful tools in biology and the unrecognized effects of chemicals can be a source of such interesting unknowns. Indeed, we identified some vectors that were not associated with an existing body of knowledge, such as GO, even though the chemical structures of the high-scoring compounds were similar (Supplementary Data). With respect to chemical biology, we believe that our decomposition strategy is appropriate for understanding the properties of natural products, whose effects are often complicated and complex^40^. Although the elucidation of such possibilities is not within the scope of this study, it is an attractive challenge for the future.

In this study, we have identified unrecognized effects of five drugs, focusing on ER-stress inducers. We believe that the “decomposition and understanding” strategy using OLSA is useful to illuminate such latent effects of chemicals and will lead us to a deeper understanding of the MOA of chemicals, which could contribute to drug discovery and chemical biology.

## Methods

### Materials

Anti-IRE1α (pSer724, Phosphorylated, Rabbit-Poly, #NB100-2323) was purchased from Funakoshi Co., Ltd. (Tokyo, Japan). Anti-GRP78 (KDEL monoclonal antibody, 10C3, #ADI-SPA-827-D) was purchased from Enzo Life Sciences (Farmingdale, NY). Anti-β-actin (#sc-47778) was purchased from Santa Cruz Biotechnology (Dallas, TX). D-cycloserine (#034-21001) and sodium chloride (#191-01665) were purchased from Fujifilm Wako Pure Chemical Corporation (Osaka, Japan). Ciclosporin A (#C2408), cyproheptadine hydrochloride sesquihydrate (#C3218), and perphenazine (#P1970) were purchased from Tokyo Chemical Industry Co., Ltd (Tokyo, Japan). Trimipramine maleate (#HY-B1213) and protriptyline hydrochloride (#HY-B0949) were purchased from Namiki Shoji Co., Ltd. (Tokyo, Japan). Phenoxybenzamine (#B019-250MG) was purchased from Sigma-Aldrich (St. Louis, MO). Ciclopirox (#A10213-10) was purchased from AdooQ BioScience (Irvine, CA). Delsoline (#sc-252666) was purchased from Santa Cruz.

### Cell culture

MCF7 cells and HuH7 cells were cultured in high-glucose Dulbecco’s modified Eagle’s Medium (DMEM) (#11995-073, Thermo Fisher Scientific, Waltham, MA) supplemented with 10% FBS. HepG2 cells were cultured in DMEM with 10% FBS and 1% MEM non-essential amino acids (#11140-050, Life Technologies, Waltham, MA). PXB cells (#PPC-P02) and culture medium (#PPC-M100) were purchased from PhoenixBio (Hiroshima, Japan). All cells were maintained at 37 °C under 5% CO_2_.

### XBP1 splicing test

After a 6-h treatment with compounds, the cells were washed with PBS and cDNA was prepared with SuperPrep II Cell Lysis & RT Kit for qPCR (Toyobo, Osaka, Japan). Note that the wash process removes the dead cells observed under some conditions. Synthesized cDNA was amplified by conventional PCR with KOD One PCR Master Mix (Toyobo); 25 cycles of denaturation: 98 °C, 10 s; annealing: 60 °C, 5 s; extension: 68 °C, 1 s using the following primers (hXBP1 Fw: GAGTTAAGACAGCGCTTGGG, Rv: ACTGGGTCCAAGTTGTGCAG, mXbp1 Fw: GAGTTAAGACAGCGCTTGGG, Rv: ACTGGGTCCAAGTTGTGCAG). Each DNA concentration was determined using MultiNA (electrophoresis apparatus incorporating a microchip). Band density was quantified with the pre-installed software of MultiNA. Values were calculated as the ratio of concentrations of spliced to unspliced XBP1. Raw data are summarized in Supplementary Data.

### Western blotting analysis

Western Blotting analysis was conducted as previously described^7^. Briefly, specimens were separated with sodium dodecyl sulphate polyacrylamide gel electrophoresis on a 10% polyacrylamide gel with a 3.75% stacking gel at 140 V for 90 min. The molecular weight was determined using Precision Plus Protein Standards (#1610373, Bio-Rad, Richmond, CA). Proteins were transferred electrophoretically to a poly(vinylidene difluoride) (PVDF) membrane (Pall, Port Washington, NY) using a blotter (Bio-Rad) at 100 V for 60 min. Non-specific binding sites on the membrane were blocked with PVDF Blocking Reagent for Can Get Signal (Toyobo) at room temperature for 60 min. After blocking, the PVDF membrane was incubated with primary antibodies diluted with Can Get Signal solution 1 (Toyobo) at 4 °C for 24 h. Primary antibodies were used as follows: anti-β-actin (1/2,000), anti-p-IRE1α (1/2,000), and anti-GRP78 (1/1,000).

After the reaction with primary antibodies, the membrane was incubated with horseradish peroxidase-conjugated anti-rabbit or anti-mouse IgG antibody (Amersham Biosciences, Piscataway, NJ) diluted to 1/10,000 in Tris-buffered saline containing 0.05% Tween 20 at room temperature for 60 min. Immunoreactivity was detected with a Fusion Solo S (Vilber Lourmat, Marne-la-Vallée, France) and Westar ETA C Ultra 2.0 (Cyanagen, Bologna, Italy). The band intensity indicating each protein was quantified by FusionCapt Advance solo 7 software (Vilber Lourmat). Uncropped blots are summarized in Supplementary Fig. S14.

### Quantitative PCR

Total RNA was extracted, and cDNA was reverse transcribed with SuperPrep II Cell Lysis & RT Kit for qPCR (Toyobo). Quantitative PCR analysis was performed using Thunderbird Probe qPCR Mix (Toyobo) and a LightCycler 480 Instrument II (Nippon Genetics Co, Ltd., Tokyo, Japan). Levels of mRNA for each gene of interest were normalized to levels of mRNA for human glyceraldehyde 3-phosphate dehydrogenase (GAPDH). OPC grade PCR primers were purchased from Eurofins Genomics (Tokyo, Japan), with sequences as follows:

hGAPDH (Fw: GGGGAGCCAAAAGGGTCATCATCT, Rv: GACGCCTGCTTCACCACCTTCTTG), hGRP78 (Fw: CACGGTCTTTGACGCCAAG, Rv: CCAAATAAGCCTCAGCGGTTT), hCHOP (Fw: GAACGGCTCAAGCAGGAAATC, Rv: TTCACCATTCGGTCAATCAGAG).

Raw data are summarized in Supplementary Data.

### Profile data analysis

Based on previous applications of OLSA, we focused on gene expression changes accounting for 80% of the cumulative contribution and obtained 118 individual effects of drugs, represented as vectors composed of genes (gene signatures). Here, the vectors were named according to their explanation of the variance in whole gene expression as a vector called P”*X*”V where X indicates the *X*th highest variance. GO analysis was conducted using Gene Ontology Consortium (https://geneontology.org/) with the following settings: analysis type: PANTHER Overrepresentation Test (released 2019-06-06); Annotation Version and Release Date: GO Ontology database released 2019-02-02; Analysed List:upload_1 (Homo sapiens); Reference List: Homo sapiens (all genes in database); Test Type: FISHER; and Correction: FDR. Hierarchical clustering is with the word method; Euclid distance was conducted using the Scipy library of Python 3.

### Statistical analysis

Welch’s t test and permutation test were used to identify significant differences between groups, where appropriate. The data were analysed using the statsmodels and Scikit-learn libraries of Python 3.

## Supplementary information

Supplementary information 1.

Supplementary information 2.

## Data Availability

The computer code used in this study is available in the following database: OLSA python scripts: GitHub (https://github.com/mizuno-group/OLSApy). CMap data: https://www.ilincs.org/ilincs/signatures/main. Gene Expression Omnibus: https://www.ncbi.nlm.nih.gov/geo/.
