# Supplementary material for: Decomposition profile data analysis of multiple drug effects identifies endoplasmic reticulum stress-inducing ability as an unrecognized factor
Source: Sci Rep. 2020 Aug 4;10:13139. doi: 10.1038/s41598-020-70140-9 (PMC7403579; doi:10.1038/s41598-020-70140-9)
Supplement: Supplementary file 1 — Supplementary information 1. [file 41598_2020_70140_MOESM1_ESM.docx]

**Title**

**Decomposition Profile Data Analysis of Multiple Drug Effects Identifies Endoplasmic Reticulum Stress-Inducing Ability as an Unrecognized Factor**

**Katsuhisa Morita^†, 1^, Tadahaya Mizuno^†,^ *^, 1^, and Hiroyuki Kusuhara**^, 1^**

^1^Graduate School of Pharmaceutical Sciences, the University of Tokyo, Bunkyo-ku, Tokyo, 113-0033, Japan

† equally contributed to this work

* Corresponding author: Tel: +81-3-5841-4771; E-mail: [tadahaya@mol.f.u-tokyo.ac.jp](mailto:tadahaya@mol.f.u-tokyo.ac.jp)

** Corresponding author: Tel: +81-3-5841-4770; E-mail: [kusuhara@mol.f.u-tokyo.ac.jp](mailto:kusuhara@mol.f.u-tokyo.ac.jp)

**Supplementary Figure**


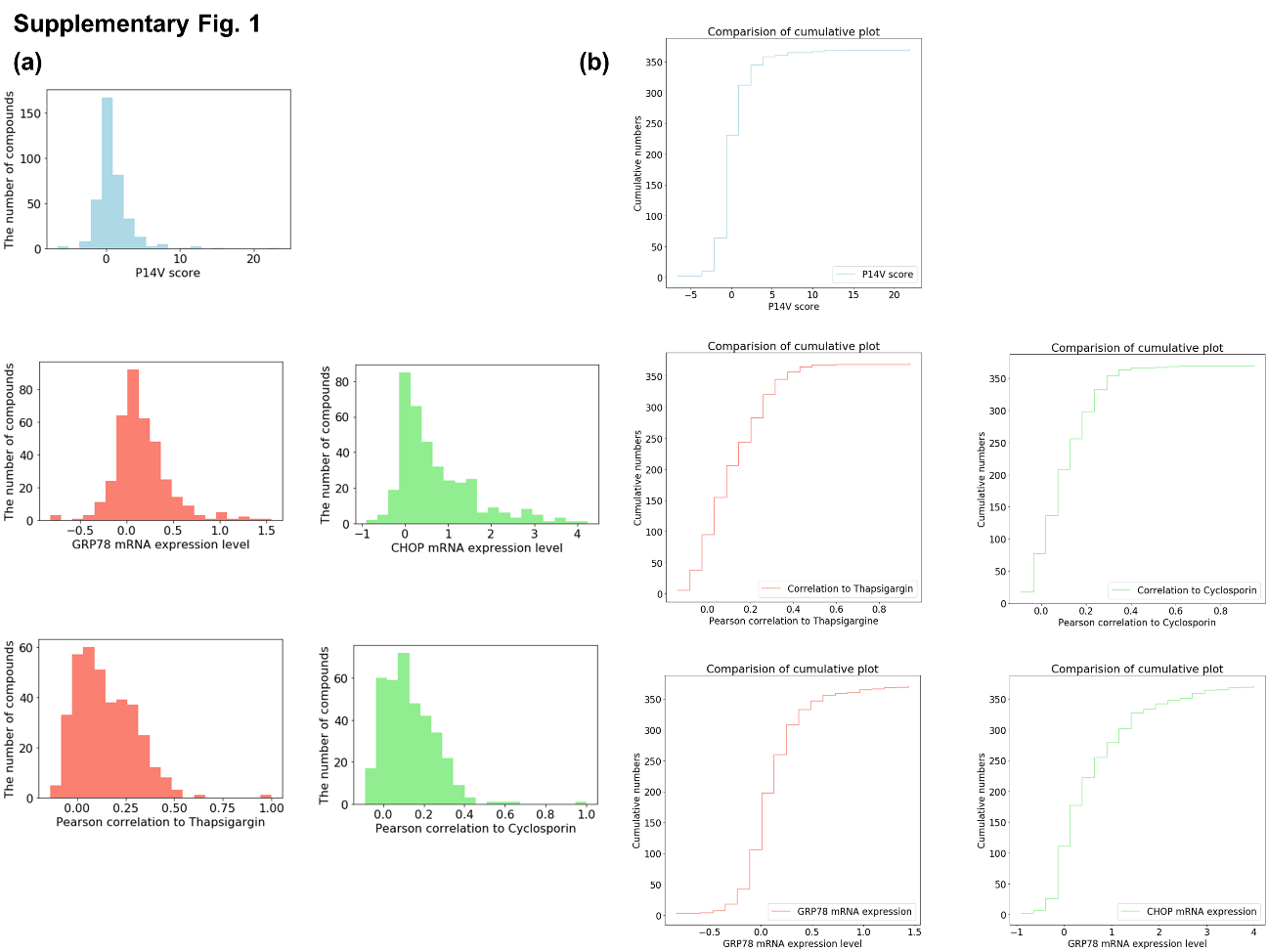


**Supplementary Fig. S1 Distribution of the scores of each estimator**

a. Frequency distribution of the scores. Histograms of all compounds in the dataset. Horizontal axes indicate P14V score, GRP and CHOP mRNA expression of transcriptome, and Pearson correlation to thapsigargin and cyclosporin.

b. Cumulative frequency distribution of the scores of all compounds in the dataset. Horizontal axes indicate the same with Supplementary Fig. S1 (a).


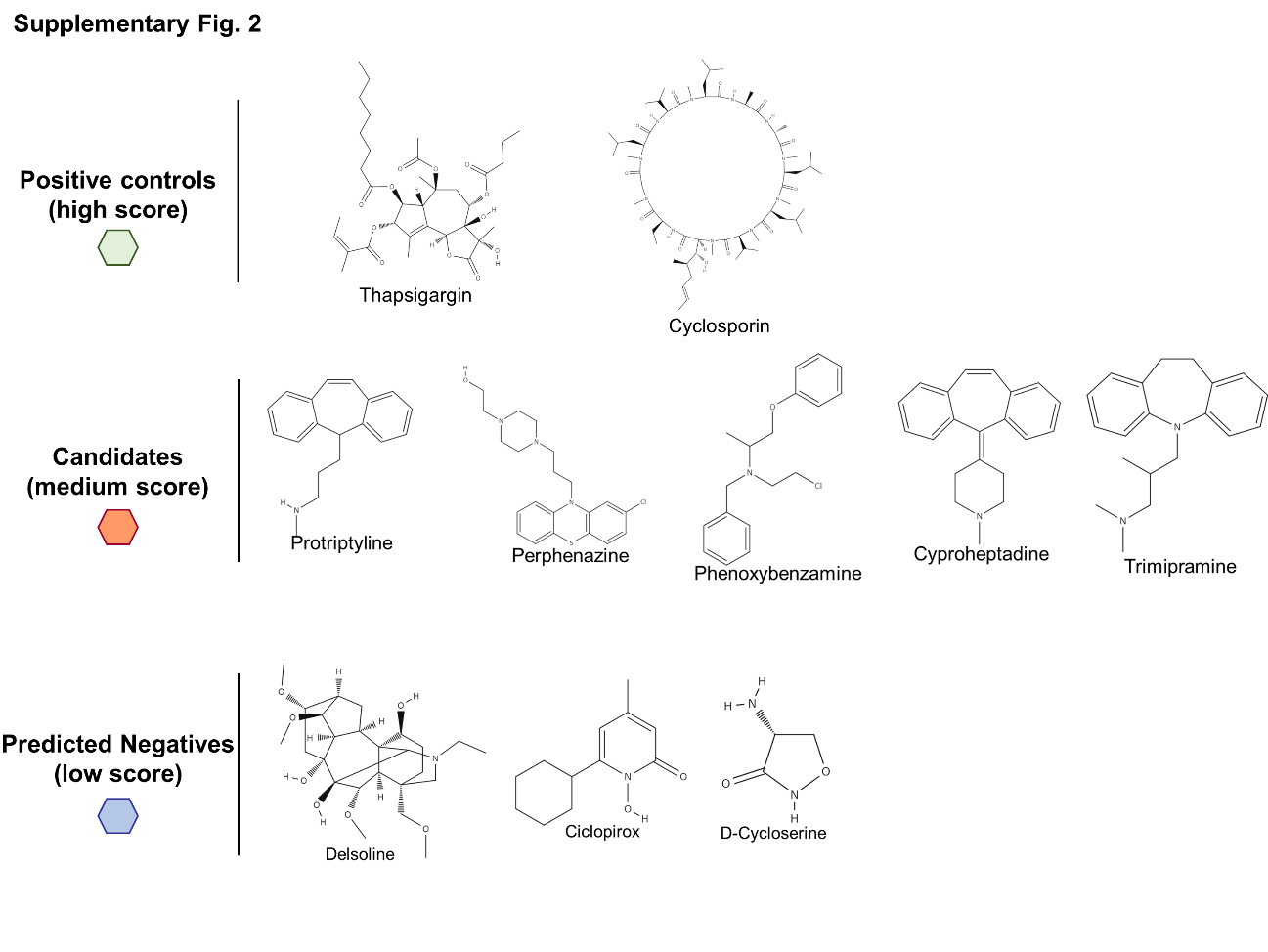


**Supplementary Fig. S2 Structure of the candidate drugs and the control chemicals**

Structural formulas of positive controls, medium score candidates, and predicted negative compounds.


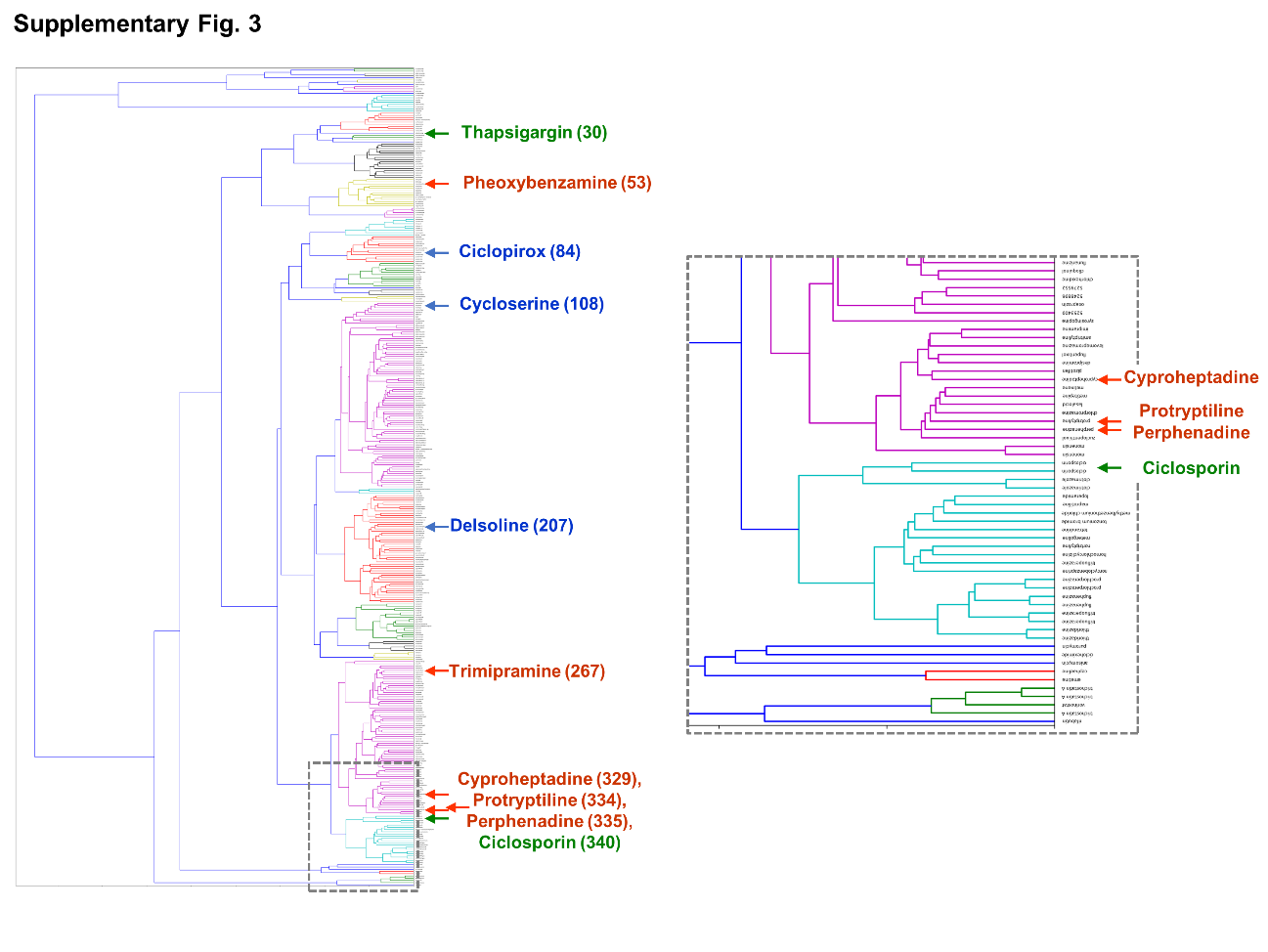


**Supplementary Fig. S3 Unsupervised clustering analysis**

Dendrogram of the result of hierarchical clustering of all compounds in the dataset. Ward method was employed. An area some of the candidates enriched is enlarged and shown in the right side.


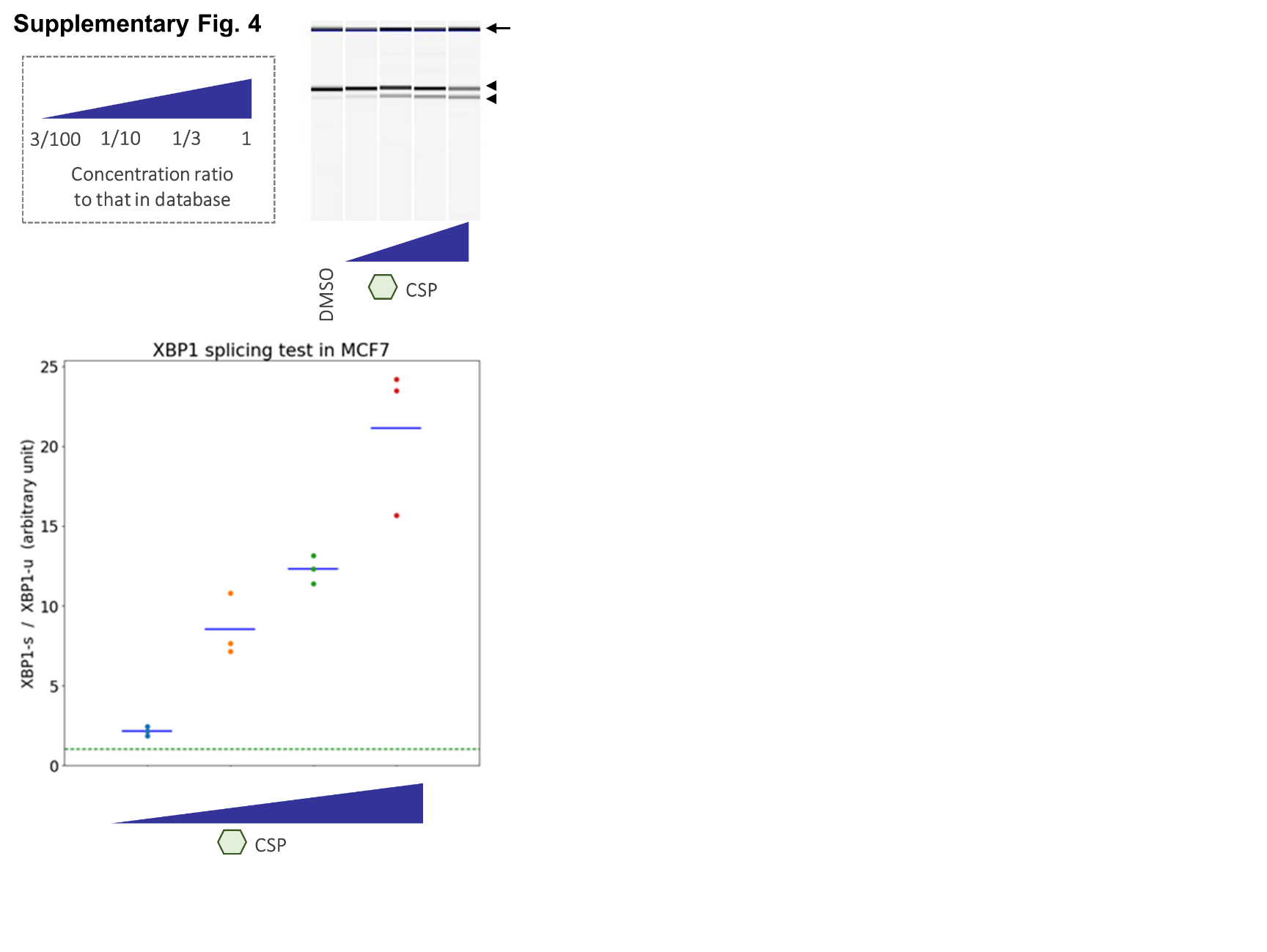


**Supplementary Fig. S4 Dynamic range of XBP1 test**

MCF7 cells were treated with various concentration of cyclosporin for 6 hours (3/100, 1/10, 1/3, and 1 times concentration in the dataset). cDNA was synthesized from mRNA and subjected to conventional PCR. The concentrations of spliced and unspliced XBP1 amplified products were quantified using a MultiNA electrophoresis apparatus. The arrow, the upper arrowhead, and the lower arrowhead indicate a non-specific band, the unspliced XBP1, and the spliced XBP1, respectively. Each value in the lower graph indicates a ratio of spliced to unspliced XBP1 concentration. CSP, ciclosporin A.


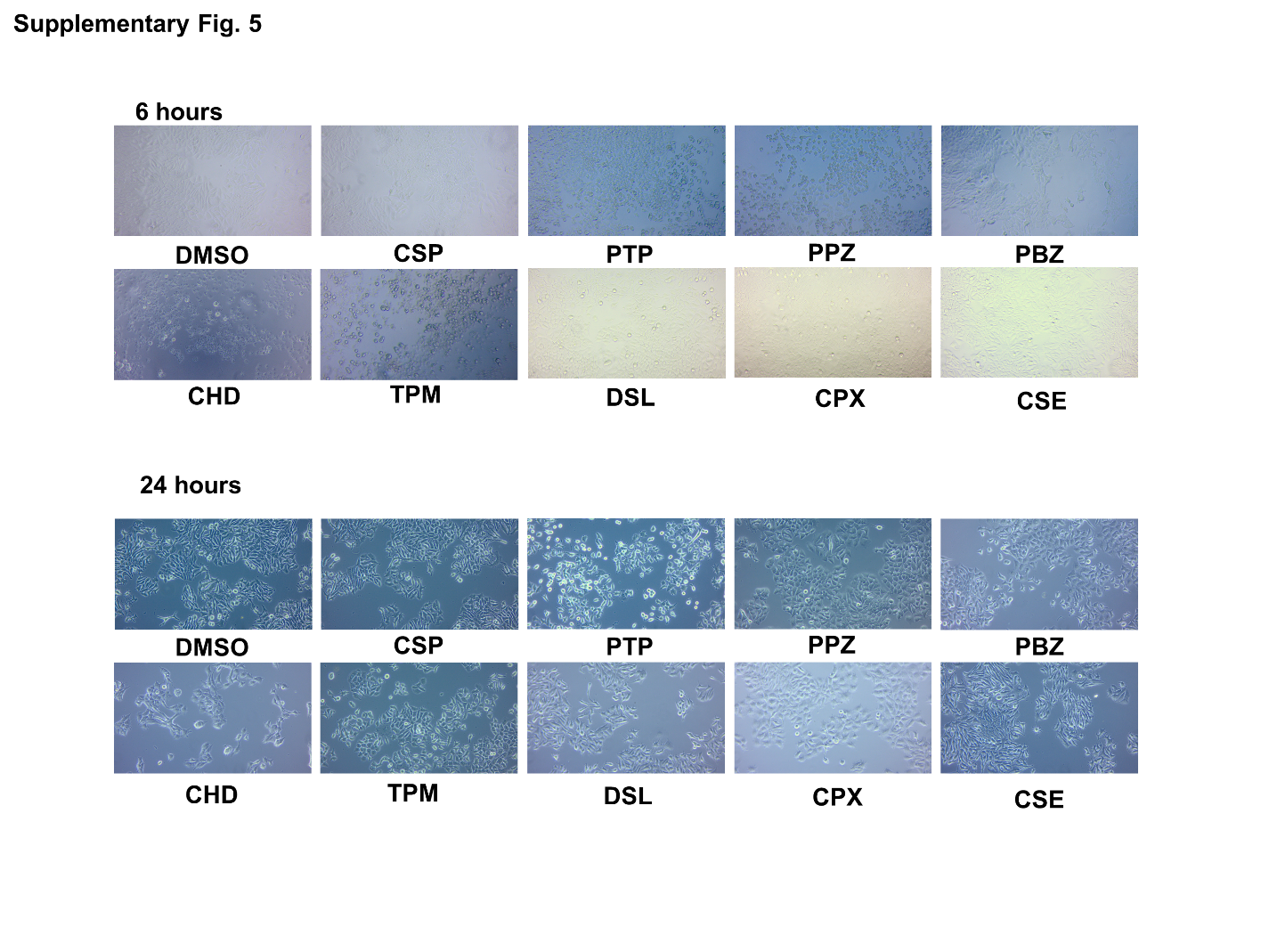


**Supplementary Fig. S5 Morphology of MCF7 cells treated with the candidates at high concentration for 6 h and 24 h**

MCF7 cells were treated with the candidate drugs for 6 h and 24 h at X-times and Y-times the concentration used for the acquisition of transcriptome data, respectively. X: CSP, 1; PTP, 3; PPZ, 3; PBZ, 10; CHD, 10; TPM, 10; DSL, 10; CSP, 10; CSE, 10. Y: CSP, 1; PTP, 2; PPZ, 1; PBZ, 10; CHD, 3; TPM, 5; DSL, 10; CPX, 10; CSE, 10. After treatment, cells were pictured with a microscopy by 10 times magnification.


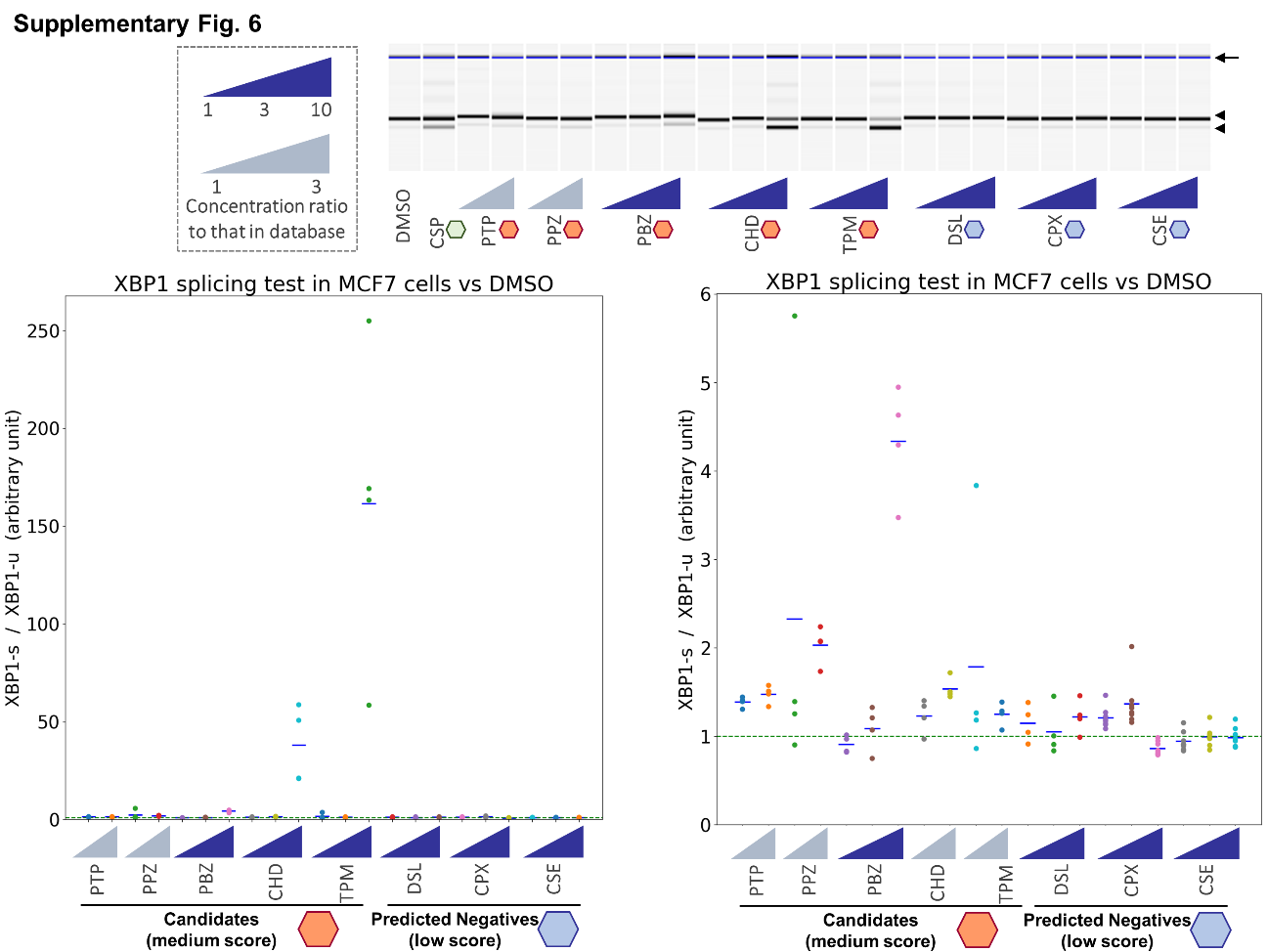


**Supplementary Fig. S6 XBP1 splicing test of MCF7 cells treated with chemicals at various concentrations**

MCF7 cells were treated with the candidate drugs for 6 h at X-times the concentration used for the acquisition of transcriptome data. X is indicated in the dashed box in the figure. cDNA was synthesized from mRNA and subjected to conventional PCR. The concentrations of spliced and unspliced XBP1 amplified products were quantified using a MultiNA electrophoresis apparatus. The arrow, the upper arrowhead, and the lower arrowhead indicate a non-specific band, the unspliced XBP1, and the spliced XBP1, respectively. Each value in the lower graph indicates a ratio of spliced to unspliced XBP1 concentration.


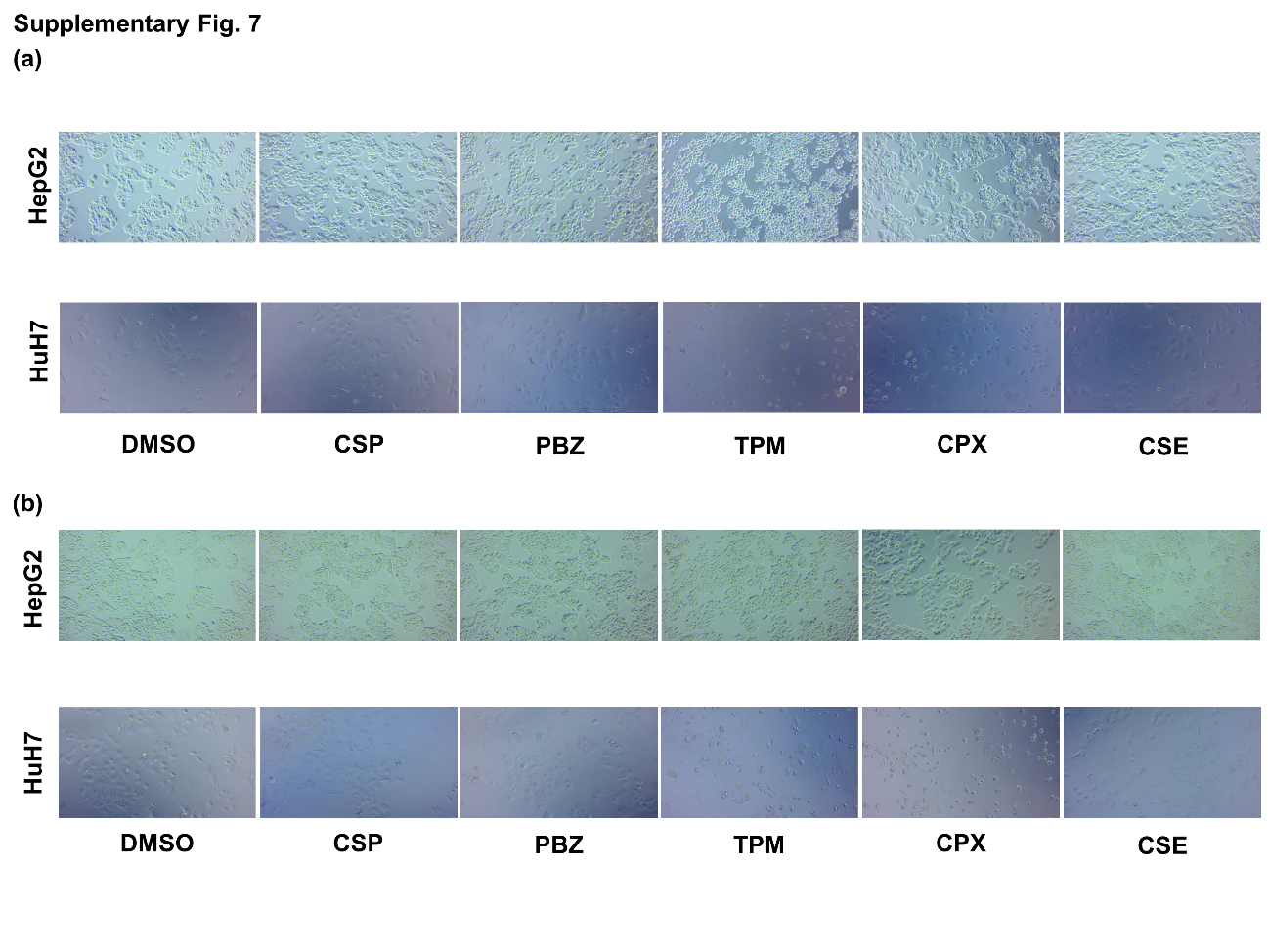


**Supplementary Fig. S7 Morphology of HepG2 cells and HuH7 cells treated with the candidates at high concentration**

a, b. HepG2 and HuH7 cells were treated with the candidate drugs for 6 h and 24 h at X-times and Y-times the concentration used for the acquisition of transcriptome data, respectively. X: CSP, 1; PBZ, 10; TPM, 10; CPX, 10; CSE, 10. Y: CSP, 1; PBZ, 3; TPM, 3; CPX, 10; CSE, 10. After treatment, cells were pictured with microscopy by 10 times magnification.


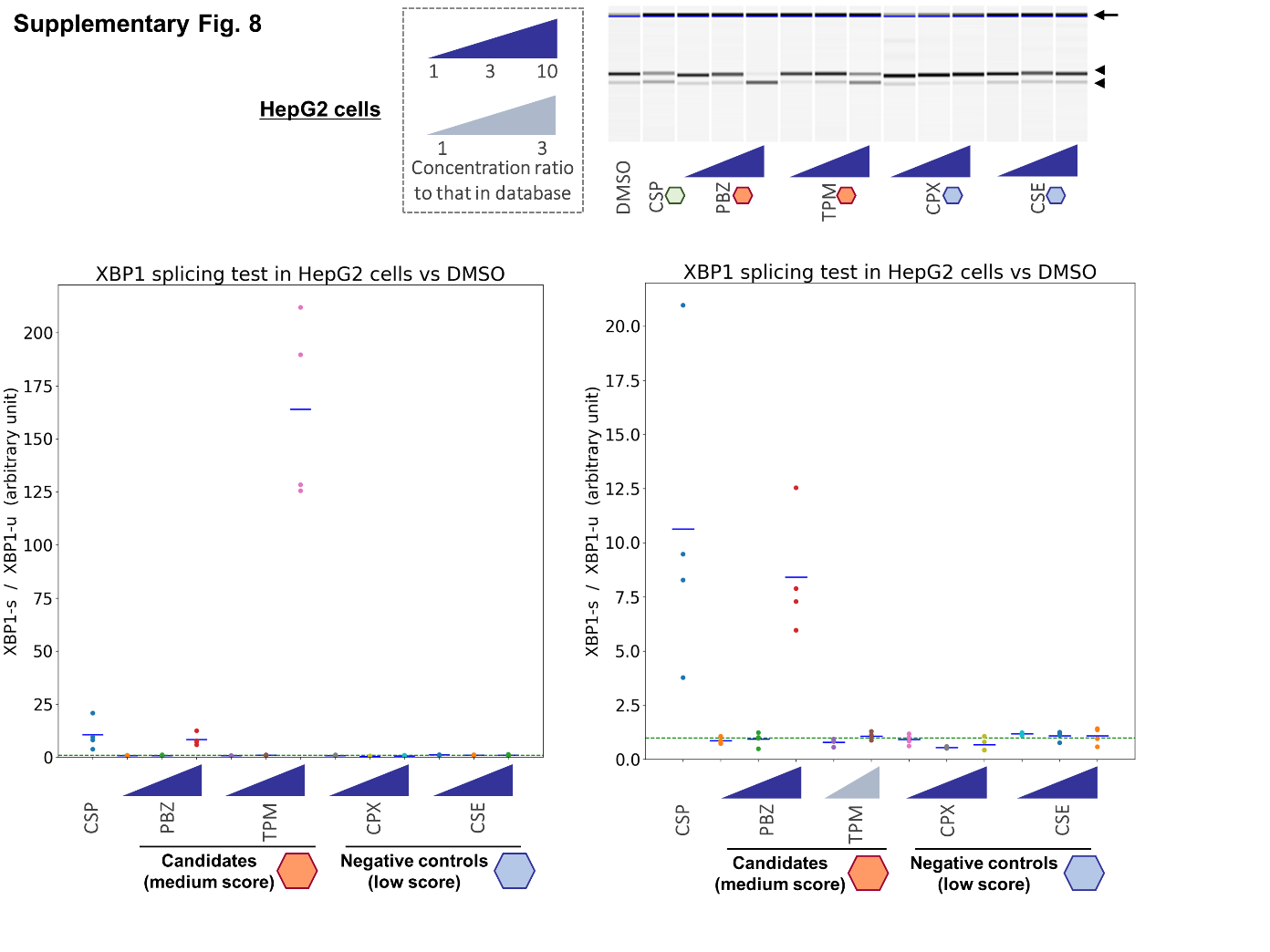


**Supplementary Fig. S8** **XBP1 splicing test of HepG2 cells treated with chemicals at various concentrations**

HepG2 cells were treated with the candidate drugs for 6 h at X-times the concentration used for the acquisition of transcriptome data. X is indicated in the dashed box in the figure. cDNA was synthesized from mRNA and subjected to conventional PCR. The concentrations of spliced and unspliced XBP1 amplified products were quantified using a MultiNA electrophoresis apparatus. The arrow, the upper arrowhead, and the lower arrowhead indicate a non-specific band, the unspliced XBP1, and the spliced XBP1, respectively. Each value in the lower graph indicates a ratio of spliced to unspliced XBP1 concentration.


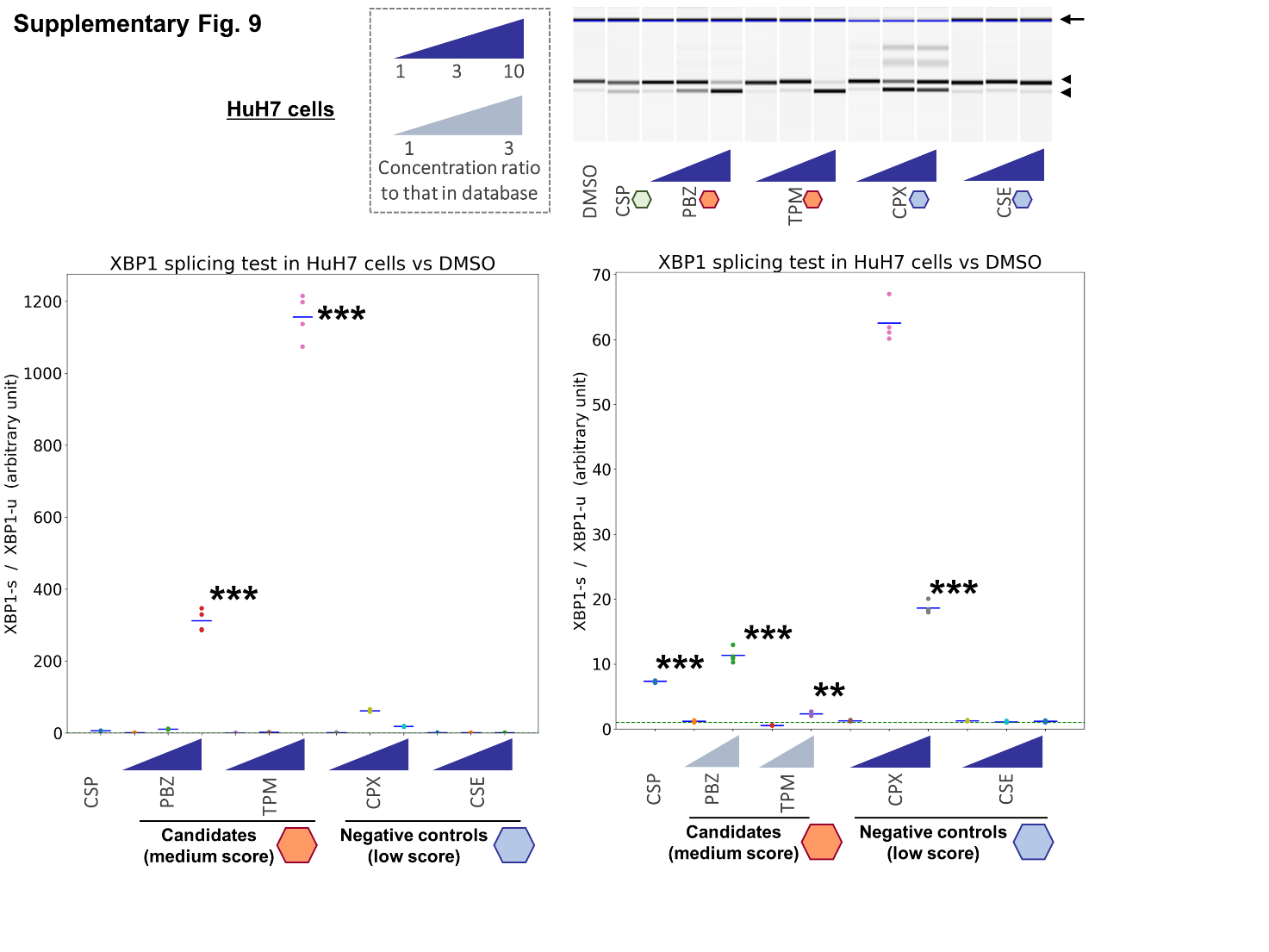


**Supplementary Fig. S9** **XBP1 splicing test of HuH7 cells treated with chemicals at various concentrations**

HuH7 cells were treated with the candidate drugs for 6 h at X-times the concentration used for the acquisition of transcriptome data. X is indicated in the dashed box in the figure. cDNA was synthesized from mRNA and subjected to conventional PCR. The concentrations of spliced and unspliced XBP1 amplified products were quantified using a MultiNA electrophoresis apparatus. The arrow, the upper arrowhead, and the lower arrowhead indicate a non-specific band, the unspliced XBP1, and the spliced XBP1, respectively. Each value in the lower graph indicates a ratio of spliced to unspliced XBP1 concentration.


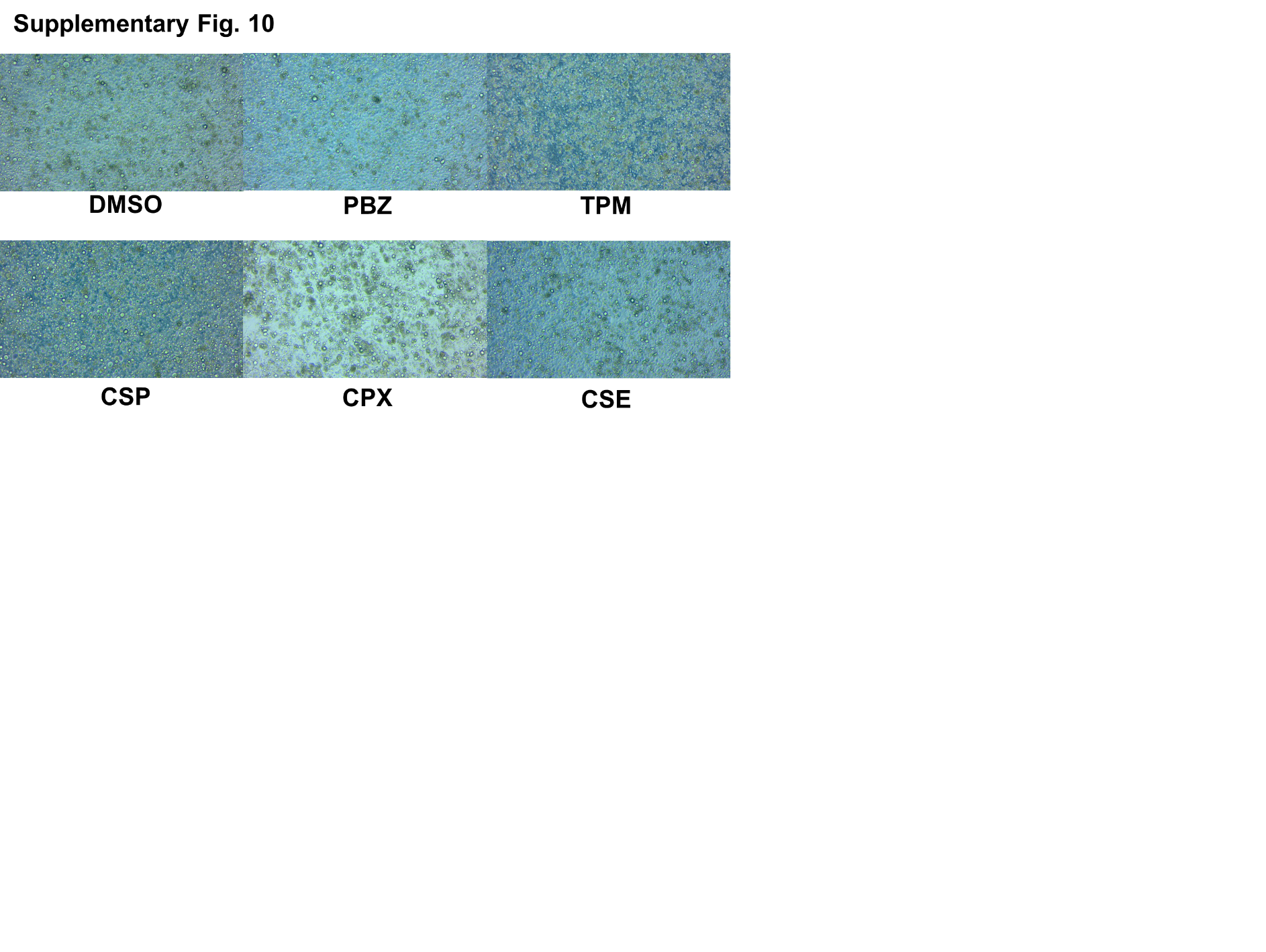


**Supplementary Fig. S10** **Morphology of PXB cells treated with the candidates at high concentration**

PXB cells were treated with the candidate drugs for 24 h at X-times the concentration used for the acquisition of transcriptome data. X: CSP, 1; PBZ, 10; TPM, 10; CPX, 10; CSE, 10. After treatment, cells were pictured with microscopy by 10 times magnification.


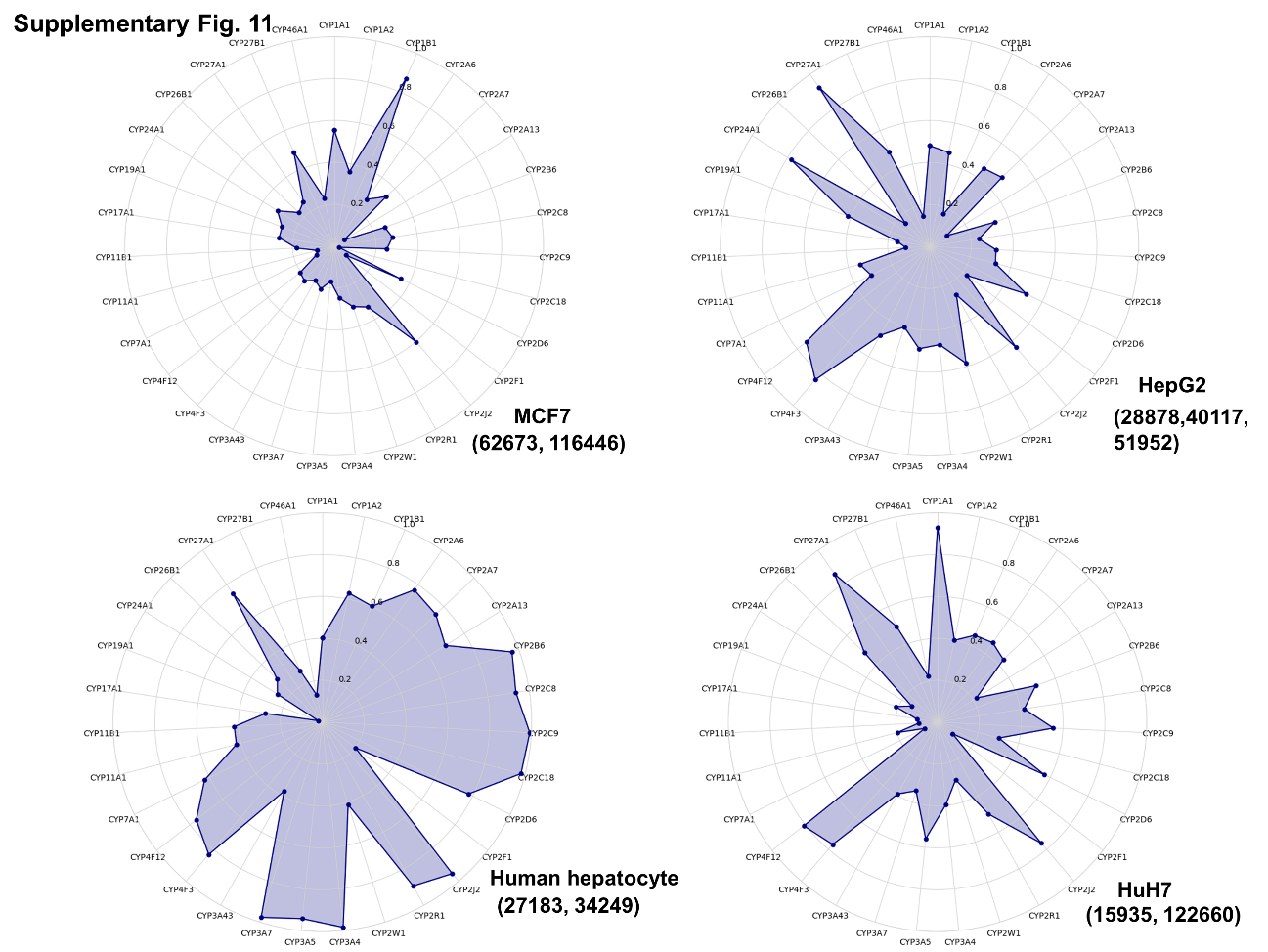


**Supplementary Fig. S11** **Expression profiles of CYP enzymes**

Radar chart visualizing mRNA expression of cytochrome P450 (CYP) enzymes collected from transcriptome data. Transcriptome data were obtained from Gene Expression Omnibus and converted to expression ranking in descending order. Ranking was divided by the number of genes in each data to normalize the data and medians of the normalized ranking were shown. The number at bottom right of each graph indicates GSE number.


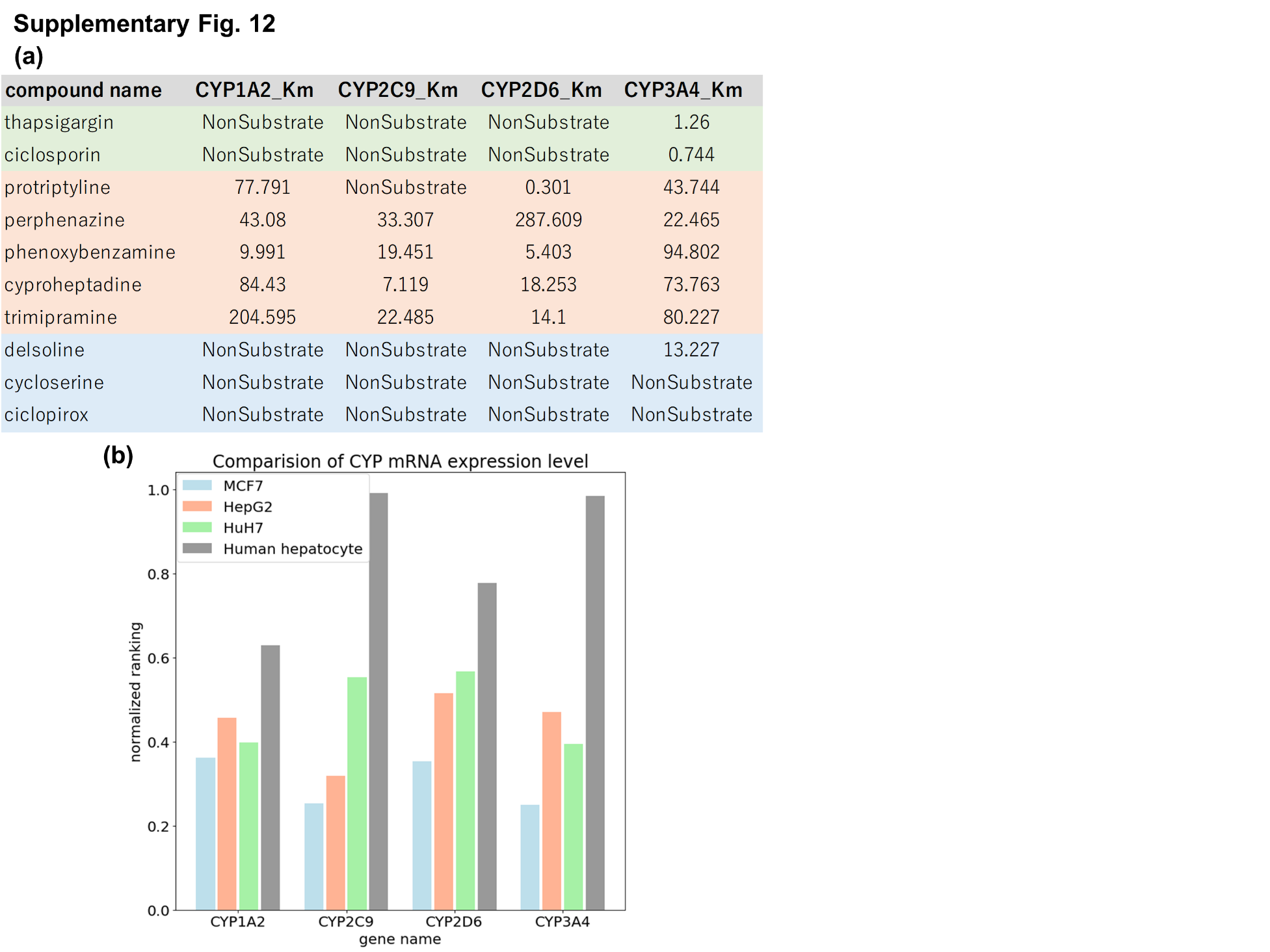


**Supplementary Fig. S12** **Information of CYP enzymes related to the tested compounds**

a. Km values of representative CYP enzymes to the tested compounds. Estimated Km values were calculated with ADMET Predictor®.

b. Comparison of mRNA expression level of CYP enzymes related to the tested compounds. Normalized ranking of CYP1A2, CYP2C9, CYP2D6, and CYP3A4 in each cell line were extracted and visualized as bar graph.


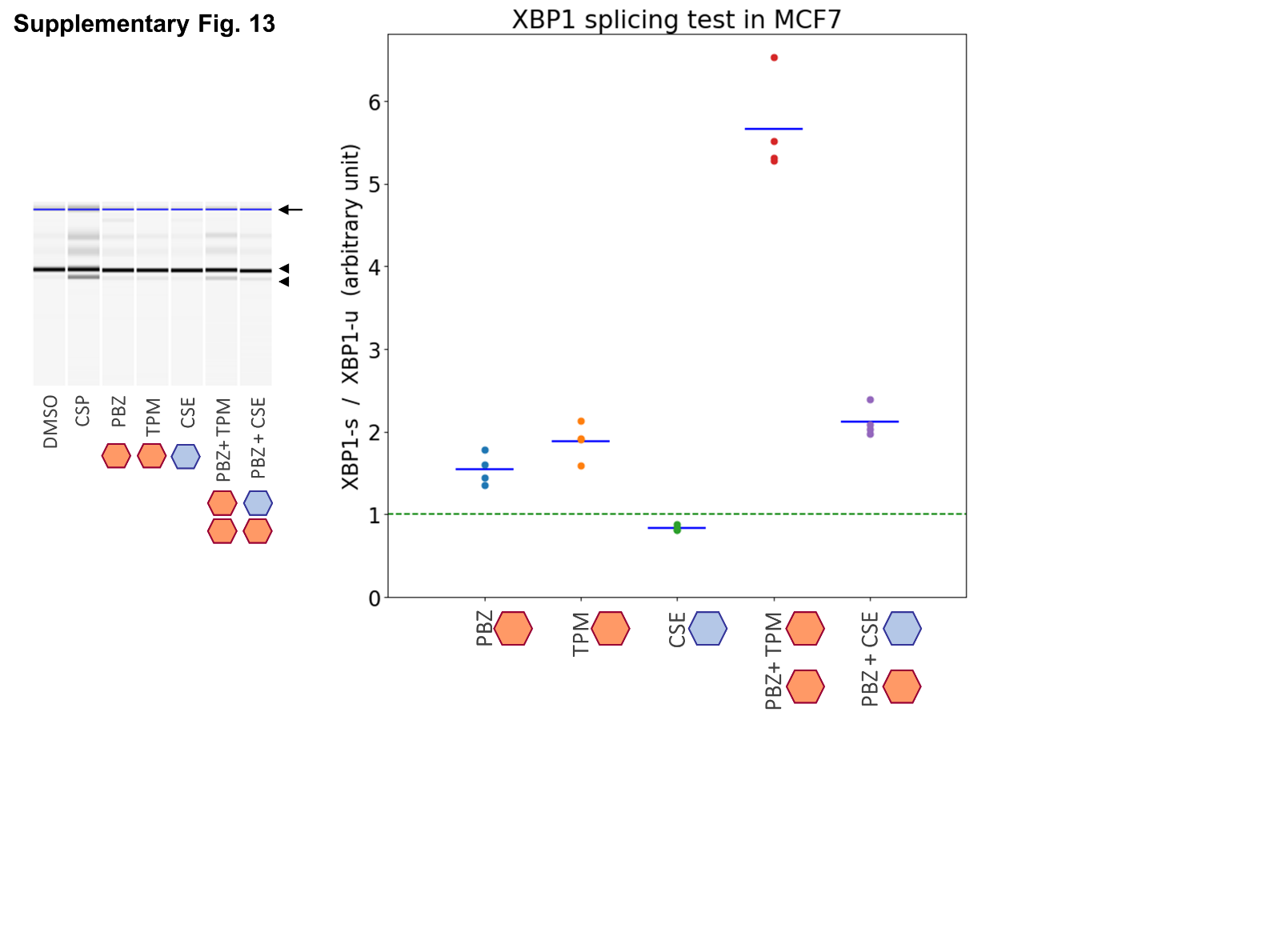


**Supplementary Fig. S13** **XBP1 splicing test of MCF7 cells co-treated with phenoxibenzamine and trimipramine**

MCF7 cells were treated with single or combinatorial treatment of the candidate drugs for 6 h at X-times the concentration used for the acquisition of transcriptome data. X: CSP, 1; PBZ, 3; TPM, 3; CSE, 10. cDNA was synthesized from mRNA and subjected to conventional PCR. The concentrations of spliced and unspliced XBP1 amplified products were quantified using a MultiNA electrophoresis apparatus. The arrow, the upper arrowhead, and the lower arrowhead indicate a non-specific band, the unspliced XBP1, and the spliced XBP1, respectively. Each value in the lower graph indicates a ratio of spliced to unspliced XBP1 concentration.


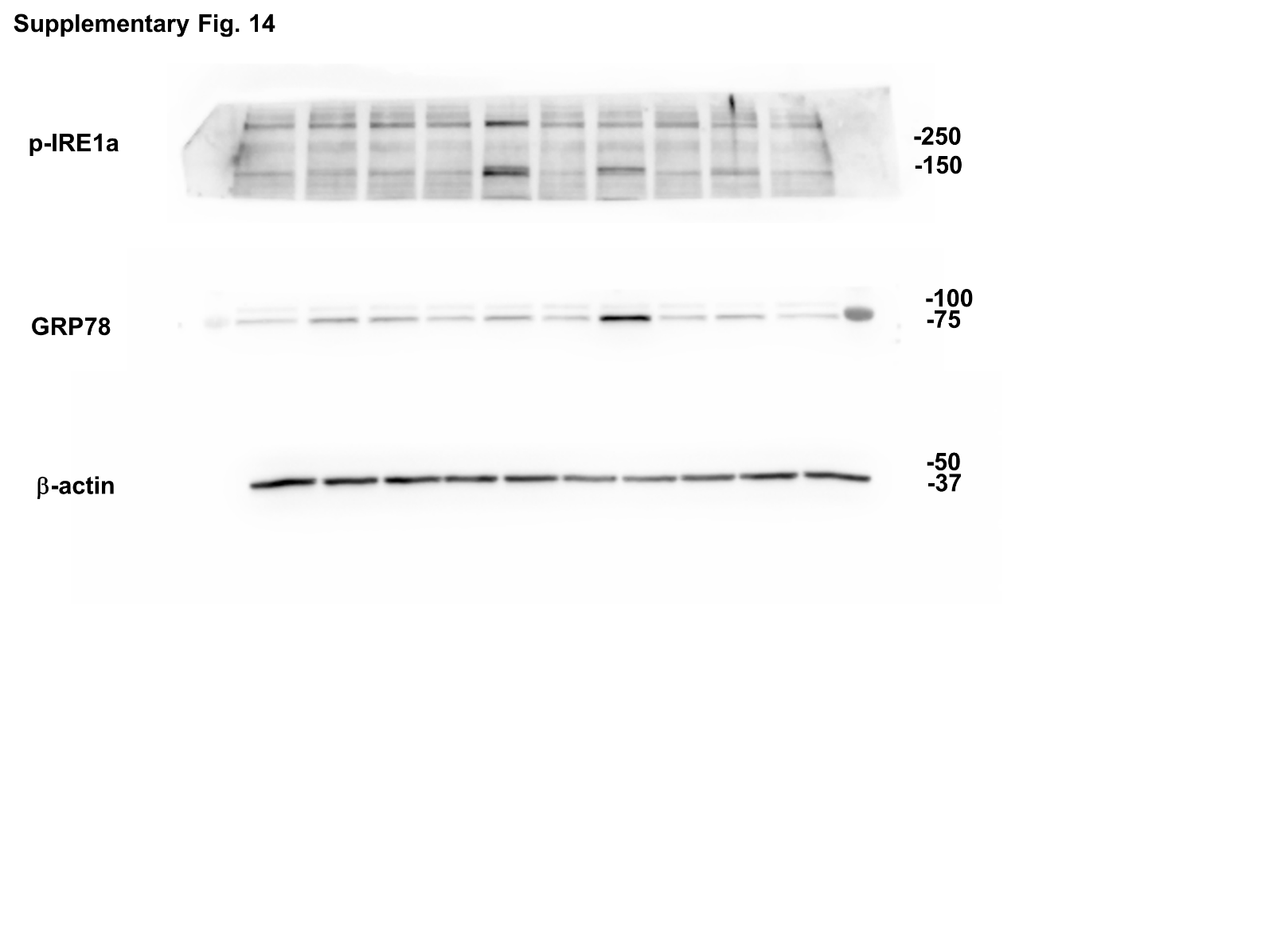


**Supplementary Fig. S14** **Uncropped images of western blotting results**

Uncropped images of western blotting results shown in Figure 3.
